# Supplementary material for: WNT signalling promotes NF-κB activation and drug resistance in KRAS-mutant colorectal cancer
Source: EMBO Rep. 2025 Nov 4;26(23):5728–55. doi: 10.1038/s44319-025-00588-1 (PMC12678608; doi:10.1038/s44319-025-00588-1)
Supplement: Supplementary file 1 — Table EV1 [file 44319_2025_588_MOESM1_ESM.pdf]

Table EV1: summary of statistics.

P valµe style: 0.12 (ns), 0.033(*), 0.002(**), <0.001 (***)

Figure1A	mean 1	mean 2	mean Diff	SE of Diff	n1	n2	Summary
Control_DMSO vs. RasG12V_DMSO	102.6	31.77	70.84	7.236	18	42	***
Control_DMSO vs. RasG12V_1µM Trametinib	102.6	84.44	18.17	7.319	18	39	ns
RasG12V_DMSO vs. RasG12V_Trametinib	31.77	84.44	-52.67	5.712	42	39	***
RAP vs. RAP_Trametinib	27.81	40.31	-12.5	6.018	27	56	ns
Control_DMSO vs. RAP_Trametinib	102.6	27.81	74.8	7.816	18	27	***

Figure1B		2−ΔΔCt	
Control	1	1	1
RAP	85.83	73.43	61.68

Figure1C	mean 1	mean 2	mean Diff	SE of Diff	n1	n2	Summary
RAP+dif-RNAi_DMSO vs. RAP+dif-RNAi_Trametinib	2.778	35.93	-33.15	12.39	9	9	*
RAP+dl-RNAi_DMSO vs. RAP+dl-RNAi_Trametinib	5.845	42.71	-36.86	10.29	11	16	**

Figure1D	mean 1	mean 2	mean Diff	SE of Diff	n1	n2	Summary
Control_DMSO vs. Control+dif-RNAi_DMSO	103.6	118.5	-14.9	16.68	17	10	ns
Control_DMSO vs. Control+dif-RNAi_Trametinib	103.6	92.42	11.23	16.68	17	10	ns
Control_DMSO vs. Control+dl-RNAi_DMSO	103.6	107.5	-3.811	14.58	17	16	ns
Control_DMSO vs. Control+dl-RNAi_Trametinib	103.6	114.5	-10.84	15.42	17	13	ns

Figure1E	mean 1	mean 2	mean Diff	SE of Diff	n1	n2	Summary
Ras+GFP_DMSO vs. Ras+GFP_Trametinib	14.71	95.06	-80.35	13.01	18	28	***
Ras+GFP_DMSO vs. Ras+cactRNAi_DMSO	14.71	31.09	-16.38	14.79	18	16	ns
Ras+GFP_Trametinib vs. Ras+cactRNAi_Trametinib	95.06	55.5	39.56	12.6	28	20	**
Ras+cactRNAi_DMSO vs. Ras+cactRNAi_Trametinib	31.09	55.5	-24.41	14.44	16	20	ns

Figure1F	mean 1	mean 2	mean Diff	SE of Diff	n1	n2	Summary

GFP_DMSO vs. GFP_Trametinib	100.7	114.1	-13.43	13.22	20	20	ns
cact-RNAi_DMSO vs. cact-RNAi_Trametinib	99.46	101.7	-2.202	13.59	20	18	ns

Figure1G	mean 1	mean 2	mean Diff	SE of Diff	n1	n2	Summary
RasG12V vs. ArmS10	1.183	0.99	0.1933	3.847	3	3	ns
RasG12V vs. RasG12V+ArmS10	1.183	14.55	-13.37	3.847	3	3	*
ArmS10 vs. RasG12V+ArmS10	0.99	14.55	-13.56	3.847	3	3	*

Figure1K	mean 1	mean 2	mean Diff	SE of Diff	n1	n2	Summary
RAP_DMSO vs. RAP_Trametinib	2.328	1.849	0.4798	0.2102	13	13	ns
RAP_DMSO vs. RAP+dlRNAi_trametinib	2.328	2.017	0.3119	0.2254	13	10	ns
RAP_DMSO vs. RAP+difRNAi_Trametinib	2.328	1.706	0.6221	0.2512	13	7	ns
RAP_Trametinib vs. RAP+dlRNAi_Trametinib	1.849	2.017	-0.1679	0.2254	13	10	ns
RAP_Trametinib vs. RAP+difRNAi_Trametinib	1.849	1.706	0.1423	0.2512	13	7	ns

Figure2A	mean 1	mean 2	mean Diff	SE of Diff	n1	n2	Summary
RAP+Toll1-RNAi_DMSO vs. RAP+Toll1-RNAi_Trametinib	21.39	47.93	-26.54	10.6	18	17	*
RAP+Toll9-RNAi_DMSO vs. RAP+Toll9-RNAi_Trametinib	12.24	42.71	-30.47	11.08	16	16	*

Figure2B	mean 1	mean 2	mean Diff	SE of Diff	n1	n2	Summary
Control_Trame vs. Toll1-RNAi_Trametinib	114.1	91.3	22.81	13.84	12	14	ns
Control_Trame vs. Toll9-RNAi_Trametinib	114.1	90.79	23.32	14.69	12	11	ns

Figure3C	mean 1	mean 2	mean Diff		n1	n2	Summary
RAP_trametinib vs. RAP+dl-RNAi_Trametinib	1	0.64	-0.36		14	14	*

Figure3D	mean 1	mean 2	mean Diff	SE of Diff	n1	n2	Summary
RAP+blanks-RNAi_DMSO vs. RAP+blanks-RNAi_Trametinib	16.09	45.24	-29.14	10.6	16	14	*
RAP+cht4-RNAi_DMSO vs. RAP+cht4-RNAi_Trametinib	0.7939	39.22	-38.42	9.951	18	16	***
RAP+mfs14-RNAi_DMSO vs. RAP+mfs14-RNAi_Trametinib	23.19	53.21	-30.02	9.933	17	17	**

Figure3E	mean 1	mean 2	mean Diff		n1	n2	Summary

RAP+cht5-RNAi_DMSO vs. RAP+cht5-RNAi_Trametinib	0	25	25		12	11	**

Figure3F	mean 1	mean 2	mean Diff	SE of Diff	n1	n2	Summary
Control_Trametinib vs. blanks-RNAi_Trametinib	113.6	107.8	5.835	16.03	15	12	ns
Control_Trametinib vs. cht4-RNAi_Trametinib	113.6	91.96	21.65	16.03	15	12	ns
Control_Trametinib vs. mfs14-RNAi_Trametinib	113.6	87.64	25.97	16.03	15	12	ns

Figure3H	mean 1	mean 2	mean Diff		n1	n2	Summary
RAP_trametinib vs. RAP+cht4-RNAi_Trametinib	0.905	0.6003	-0.3047		16	13	*

Figure4B	mean 1	mean 2	mean Diff	SE of Diff	n1	n2	Summary
RAP_DMSO vs. RAP_Trametinib	18.06	32.61	-14.55	7.126	20	19	ns
RAP_DMSO vs. RAP_1µM PNU-74654	18.06	16.82	1.236	7.227	20	18	ns
RAP_Trametinib vs. RAP_Trametinib+PNU-74654	32.61	59.89	-27.27	6.831	19	24	***
RAP_PNU-74654 vs. RAP_Trametinib+PNU-74654	16.82	59.89	-43.06	6.936	18	24	***

Figure4C	mean 1	mean 2	mean Diff	SE of Diff	n1	n2	Summary
RAP_DMSO vs. RAP_Trametinib	21.94	27.2	-5.254	8.091	12	34	ns
RAP_DMSO vs. RAP_10µM LF3	21.94	18.76	3.177	9.837	12	12	ns
RAP_Trametinib vs. RAP_Trametinib+LF3	27.2	42.65	-15.45	5.888	34	33	*

Figure4D	mean 1	mean 2	mean Diff	SE of Diff	n1	n2	Summary
Control_DMSO vs. Control_Trametinib +PNU-74654	103.6	104.6	-0.9748	9.758	17	11	ns
Control_DMSO vs. Control_Trametinib+10uM LF3	103.6	110.2	-6.537	9.101	17	14	ns

Figure4E	mean 1	mean 2	mean Diff	SE of Diff	n1	n2	Summary
RAP_DMSO vs. RAP_Trametinib	2.328	1.768	0.5603	0.2325	13	8	ns
RAP_DMSO vs. RAP_PNU-74654	2.328	2.274	0.05436	0.2426	13	7	ns
RAP_DMSO vs. RAP_Trametinib+PNU-74654	2.328	1.565	0.7635	0.2426	13	7	*
RAP_Trametinib vs. RAP_Trametinib+PNU-74654	1.768	1.565	0.2032	0.2678	8	7	ns

Figure5A	mean 1	mean 2	mean Diff	SE of Diff	n1	n2	Summary

CPCT006_DMSO vs. CPCT006_Trametinib	6.952	33.85	-26.89	7.411	33	26	**
CPCT006_Trametinib vs. CPCT006_Trametinib+1μM PNU-74654	33.85	46.31	-12.46	9.601	26	13	ns
CPCT006_Trametinib vs. CPCT006_Trametinib +5μM PNU-74654	33.85	83.27	-49.42	9.164	26	15	***
CPCT006_Trametinib vs. CPCT006_T +10μM PNU-74654	33.85	38.87	-5.021	9.164	26	15	ns

Figure5B	mean 1	mean 2	mean Diff	SE of Diff	n1	n2	Summary
CPCT018_Trametinib vs. CPCT018_DMSO	30.02	2.703	27.32	10.74	21	24	*
CPCT018_Trametinib vs. CPCT018_Trametinib+0.5μM PNU-74654	30.02	22.65	7.368	11.54	21	18	ns
CPCT018_Trametinib vs. CPCT018_Trametinib+1μM PNU-74654	30.02	60.32	-30.3	10.03	21	33	*
CPCT018_Trametinib vs. CPCT018_Trametinib+5μM PNU-74654	30.02	67.45	-37.43	10.54	21	26	**

Figure5C	mean 1	mean 2	mean Diff	SE of Diff	n1	n2	Summary
CPCT045_Trametinib vs. CPCT45_DMSO	63.13	19.81	43.32	10.78	24	33	***
CPCT045_Trametinib vs. CPCT045_Trametinib+5μM PNU-74654	63.13	65.1	-1.967	11.86	24	22	ns
CPCT045_Trametinib vs. CPCT045_Trametinib+10μM PNU-74654	63.13	105.9	-42.75	12.73	24	17	**
CPCT045_Trametinib vs. CPCT045_Trametinib+15μM PNU-74654	63.13	80.51	-17.37	14.2	24	12	ns

Figure5D	mean 1	mean 2	mean Diff	SE of Diff	n1	n2	Summary
CPCT050_Trametinib vs. CPCT050_DMSO	21.96	25.08	-3.119	6.352	21	21	ns
CPCT050_Trametinib vs. CPCT050_Trametinib+5μM PNU-74654	21.96	30.45	-8.488	7.448	21	12	ns
CPCT050_Trametinib vs. CPCT050_Trametinib+10μM PNU-74654	21.96	40.76	-18.8	6.431	21	20	*
CPCT050_Trametinib vs. CPCT50_Trametinib+15μM PNU-74654	21.96	24.98	-3.025	7.448	21	12	ns

Figure5E	mean 1	mean 2	mean Diff	SE of Diff	n1	n2	Summary
CPCT029_Trametinib vs. CPCT029_DMSO	19.34	1.864	17.48	4.826	28	28	**
CPCT029_Trametinib vs. CPCT029_Trametinib+0.5μM PNU-74654	19.34	13.57	5.77	6.23	28	12	ns
CPCT029_Trametinib vs. CPCT029_Trametinib+1μM PNU-74654	19.34	39.85	-20.52	5.286	28	20	***
CPCT029_Trametinib vs. CPCT029_Trametinib+5μM PNU-74654	19.34	34.63	-15.29	5.081	28	23	*

Figure5F	mean 1	mean 2	mean Diff	SE of Diff	n1	n2	Summary
RAPp1_DMSO vs. RAPp1_Trametinib	0	14.58	-14.58	9.018	7	8	ns
RAPp1_DMSO vs. RAPp1_PNU-74654	0	0	0	8.781	7	9	ns

RAPp1_DMSO vs. RAPp1_Trametinib+PNU-74654	0	23.32	-23.32	8.781	7	9	ns
RAPp1_Trametinib vs. RAPp1_Trametinib+PNU-74654	14.58	23.32	-8.733	8.467	8	9	ns
RAPp1_PNU-74654 vs. RAPp1_Trametinib+PNU-74654	0	23.32	-23.32	8.214	9	9	*
RAPp2_DMSO vs. RAPp2_Trametinib	0	14.39	-14.39	7.416	12	11	ns
RAPp2_DMSO vs. RAPp2_PNU-74654	0	7.143	-7.143	8.45	12	7	ns
RAPp2_DMSO vs. RAPp2_Trametinib+PNU-74654	0	14.58	-14.58	8.109	12	8	ns
RAPp2_Trametinib vs. RAPp2_Trametinib+PNU-74654	14.39	14.58	-0.1901	8.255	11	8	ns
RAPp2_PNU-74654 vs. RAPp2_Trametinib+PNU-74654	7.143	14.58	-7.441	9.195	7	8	ns

Figure6A	mean 1	mean 2	mean Diff	SE of Diff	n1	n2	Summary
RAP+ GFP_Trametinib+PNU-74654 vs. RAP+brm-RNAi_Trametinib+PNU-74654	1.818	55.07	-53.25	18.29	11	14	*
RAP+ GFP_Trametinib+PNU-74654 vs. RAP+shg-RNAi_Trametinib+PNU-74654	1.818	54.65	-52.83	18.6	11	13	*
RAP+ GFP_Trametinib+PNU-74654 vs. RAP+ago-RNAi_Trametinib+PNU-74654	1.818	66.52	-64.7	19.35	11	11	*
RAP+ GFP_Trametinib+PNU-74654 vs. RAP+rhoGAPp190-RNAi_Trametinib+PNU-74654	1.818	75.5	-73.68	19.83	11	10	**
RAP+ GFP_Trametinib+PNU-74654 vs. RAP+upf1-RNAi_Trametinib+PNU-74654	1.818	73.96	-72.14	21.09	11	8	**

Figure6B	mean 1	mean 2	mean Diff	SE of Diff	n1	n2	Summary
Control_Trametinib+PNU-74654 vs. brm-RNAi_Trametinib+PNU-74654	92.58	95.56	-2.989	18.77	11	12	ns
Control_Trametinib+PNU-74654 vs. shg-RNAi_Trametinib+PNU-74654	92.58	87.88	4.696	19.17	11	11	ns
Control_Trametinib+PNU-74654 vs. ago-RNAi_Trametinib+PNU-74654	92.58	106.7	-14.16	18.12	11	14	ns
Control_Trametinib+PNU-74654 vs. rhoGAPp190-RNAi_Trametinib+PNU-74654	92.58	109.1	-16.48	18.42	11	13	ns
Control_Trametinib+PNU-74654 vs. upf1-RNAi_Trametinib+PNU-74654	92.58	99.71	-7.134	18.77	11	12	ns

Figure6C	mean 1	mean 2	mean Diff	SE of Diff	n1	n2	Summary
RAP+ GFP_DMSO vs. RAP+brm-RNAi_DMSO	1.667	3.333	-1.667	5.179	12	6	ns
RAP+ GFP_DMSO vs. RAP+shg-RNAi_DMSO	1.667	3.334	-1.667	5.513	12	5	ns
RAP+ GFP_DMSO vs. RAP+ago-RNAi_DMSO	1.667	10.75	-9.083	4.435	12	10	ns
RAP+ GFP_DMSO vs. RAP+rhoGAPp190-RNAi_DMSO	1.667	6.222	-4.555	4.435	12	10	ns
RAP+ GFP_DMSO vs. RAP+upf1-RNAi_DMSO	1.667	1.667	0	5.179	12	6	ns

Figure6E	mean 1	mean 2	mean Diff	SE of Diff	n1	n2	Summary
RAP_Trametinib vs. RAP+brm-RNAi_Trametinib	5	57.47	-52.47	12.59	10	11	***

RAP_Trametinib vs. RAP+ago-RNAi_Trametinib	5	58.59	-53.59	12.59	10	11	***
RAP_Trametinib vs. RAP+shg-RNAi_Trametinib	5	29.34	-24.34	12.59	10	11	ns
RAP_Trametinib vs. RAP+upf1-RNAi_Trametinib	5	19.7	-14.7	12.59	10	11	ns
RAP_Trametinib vs. RAP+rhoGAPp190-RNAi_Trametinib	5	18.75	-13.75	12.59	10	11	ns

Figure6F	mean 1	mean 2	mean Diff	SE of Diff	n1	n2	Summary
RAP_PNU vs. RAP+rhoGAPp190-RNAi_PNU-74654	2.222	16.33	-14.11	4.524	15	20	*
RAP_PNU vs. RAP+upf1-RNAi_PNU-74654	2.222	0.5	1.722	4.524	15	20	ns
RAP_PNU vs. RAP+brm-RNAi_PNU-74654	2.222	11.81	-9.583	5.13	15	12	ns
RAP_PNU vs. RAP+ago-RNAi_PNU-74654	2.222	8.502	-6.28	5.019	15	13	ns
RAP_PNU vs. RAP+shg-RNAi_PNU-74654	2.222	1.923	0.2989	5.019	15	13	ns

Figure6G	mean 1	mean 2	mean Diff	SE of Diff	n1	n2	Summary
RAPp1_Trametinib+PNU-74654 vs. RAPp1+rhoGAPp190-RNAi_Trametinib+PNU-74654	10.97	59.71	-48.74	13.14	20	15	**
RAPp1_Ttametinib+PNU-74654 vs. RAPp1+upf1-RNAi_Trametinib+PNU-74654	10.97	78.75	-67.78	14.05	20	12	***
RAPp1_Trametinib+PNU-74654 vs. RAPp1+shg-RNAi_Trametinib+PNU-74654	10.97	35.51	-24.53	13.58	20	12	ns
RAPp1_Trametinib+PNU-74654 vs. RAPp1+ago-RNAi_Trametinib+PNU-74654	10.97	67.86	-56.89	16.89	20	7	**
RAPp1_Trametinib+PNU-74654 vs.RAPp1+brm-RNAi_Trametinib+PNU-74654	10.97	49.24	-38.27	14.44	20	11	*

Figure6H	mean 1	mean 2	mean Diff	SE of Diff	n1	n2	Summary
RAPp2_Trametinib+PNU-74654 vs. RAPp2+rhoGAPp190-RNAi_Trametinib+PNU-74654	25.45	67.98	-42.52	13.83	22	14	*
RAPp2_Trametinib+PNU-74654 vs. RAPp2+upf1-RNAi_Trametinib+PNU-74654	25.45	62.92	-37.46	13.29	22	16	*
RAPp2_Trametinib+PNU-74654 vs. RAPp2+shg-RNAi_Trametinib+PNU-74654	25.45	91.67	-66.21	14.94	22	11	***
RAPp2_Trametinib+PNU-74654 vs. RAPp2+ago-RNAi_Trametinib+PNU-74654	25.45	79.17	-53.71	14.52	22	12	**
RAPp2_Trametinib+PNU-74654 vs. RAPp2+brm-RNAi_Trametinib+PNU-74654	25.45	86.11	-60.66	14.52	22	12	***

Figure7I	mean 1	mean 2	mean Diff	SE of Diff	n1	n2	Summary
KRASMT and β-Catenin High vs. KRASControl and β-Catenin Low	84.6	54.97	29.62	9.928	28	311	**
KRASMT and β-Catenin High vs. KRASMT or β-Catenin High	84.6	52.86	31.74	10.13	28	208	**

Figure7J	mean 1	mean 2	mean Diff	SE of Diff	n1	n2	Summary
KRASMT and β-Catenin High vs. KRASControl and β-Catenin Low	115.7	115.8	-0.0841	6.808	31	291	ns

KRASMT and β-Catenin High vs. KRASMT or β-Catenin High	115.7	114.7	1.043	6.923	31	215	ns

Figure EV1A	mean 1	mean 2	mean Diff		n1	n2	Summary
Control_DMSO vs. Control_Trametinib	106.3	111.8	-5.53		12	12	ns

Figure EV1B	mean 1	mean 2	mean Diff	SE of Diff	n1	n2	Summary
Control_DMSO vs. RasG12V_DMSO	1	1.464	-0.4642	0.1141	12	13	***
Control_DMSO vs. RasG12V_Trametinib	1	1.012	-0.01153	0.1164	12	12	ns
RasG12V_DMSO vs. RasG12V_Trametinib	1.464	1.012	0.4526	0.1141	13	12	**

Figure EV1C	mean 1	mean 2	mean Diff	SE of Diff	n1	n2	Summary
Control vs. RasG12V	1	1.464	-0.4642	0.1538	15	13	*
Control vs. ArmS10	1	0.9379	0.06211	0.1777	15	8	ns
Control vs. RasG12V+ArmS10	1	1.454	-0.4537	0.1438	15	17	*
RasG12V vs. RasG12V+ArmS10	1.464	1.454	0.01041	0.1495	13	17	ns
ArmS10 vs. RasG12V+ArmS10	0.9379	1.454	-0.5158	0.174	8	17	*

Figure EV1D	mean 1	mean 2	mean Diff		n1	n2	Summary
Control vs. RasG12V	1	1.464	-0.4642	0.1456	15	13	*
Control vs. RAP	1	2.328	-1.328	0.1456	15	13	***
Control vs. RasG12V+p53RNAi	1	1.763	-0.7632	0.1403	15	15	***
RasG12V vs. RasG12V+p53RNAi	1.464	1.763	-0.2991	0.1456	13	15	ns
RasG12V vs. RAP	1.464	2.328	-0.8643	0.1507	13	13	***
RAP vs. RasG12V+p53RNAi	2.328	1.763	0.5652	0.1456	13	15	**

Figure EV2A	mean 1	mean 2	mean Diff	SE of Diff	n1	n2	Summary
RAP+Toll3-RNAi_DMSO vs. RAP+Toll3-RNAi_Trametinib	4.167	8.691	-4.524	7.585	12	12	ns
RAP+Toll4-RNAi_DMSO vs. RAP+Toll4-RNAi_Trametinib	0	0	0	10.73	6	6	ns
RAP+Toll5-RNAi_DMSO vs. RAP+Toll5-RNAi_Trametinib	0	0	0	13.14	4	4	ns
RAP+Toll6-RNAi_DMSO vs. RAP+Toll6-RNAi_Trametinib	9.775	29.03	-19.26	6.194	18	18	*
RAP+Toll7-RNAi_DMSO vs. RAP+Toll7-RNAi_Trametinib	6.666	0	6.666	12.46	5	4	ns

RAP+Toll8-RNAi_DMSO vs. RAP+Toll8-RNAi_Trametinib	12.78	4.167	8.611	7.585	12	12	ns

Figure EV2D	mean 1	mean 2	mean Diff	SE of Diff	n1	n2	Summary
RAP_Trametinib vs. RAP+Toll1RNAi_Trametinib	1.849	1.63	0.2187	0.178	13	14	ns
RAP_Trametinib vs. RAP+Toll9RNAi_Trametinib	1.849	1.703	0.1459	0.1944	13	10	ns

Figure EV3A	mean 1	mean 2	mean Diff	SE of Diff	n1	n2	Summary
RAP+rel-RNAi_DMSO vs. RAP+rel-RNAi_Trametinib	18.94	12.5	6.44	7.95	11	10	ns
RAP+bsk-RNAi_DMSO vs. RAP+bsk-RNAi_Trametinib	0	0	0	10.51	6	6	ns
RAP+dome-RNAi53890_DMSO vs. RAP+dome-RNAi53890_Trametinib	4.545	7.143	-2.597	8.797	11	7	ns
RAP+dome-RNAi34618_DMSO vs. RAP+dome-RNAi34618_Trametinib	13.33	4.762	8.571	8.137	10	10	ns

Figure EV3B	mean 1	mean 2	mean Diff	SE of Diff	n1	n2	Summary
RAP_Trametinib vs. RAP+CG9360-RNAi_Trametinib	6.19	10	-3.81	10.67	14	5	ns
RAP_Trametinib vs. RAP+ref(2)p-RNAi_Trametinib	6.19	15	-8.81	8.477	14	10	ns
RAP_Trametinib vs. RAP+CG32302-RNAi_Trametinib	6.19	5.555	0.635	9.99	14	6	ns
RAP_Trametinib vs. RAP+CG17104-RNAi_Trametinib	6.19	17.71	-11.52	9.074	14	8	ns
RAP_Trametinib vs. RAP+mec2-RNAi_Trametinib	6.19	15.83	-9.643	8.477	14	10	ns
RAP_Trametinib vs. RAP+CG1698-RNAi_Trametinib	6.19	0	6.19	10.67	14	5	ns
RAP_Trametinib vs. RAP+CG15739-RNAi_Trametinib	6.19	11.11	-4.921	8.748	14	9	ns
RAP_Trametinib vs. RAP+ag5r-RNAi_Trametinib	6.19	7.575	-1.385	8.249	14	11	ns
RAP_Trametinib vs. RAP+arc1-RNAi_Trametinib	6.19	13.89	-7.698	9.99	14	6	ns
RAP_Trametinib vs. RAP+CG2065-RNAi_Trametinib	6.19	0	6.19	9.99	14	6	ns
RAP_Trametinib vs. RAP+CG10182-RNAi_Trametinib	6.19	6.25	-0.06	9.074	14	8	ns
RAP_Trametinib vs. RAP+CG18473-RNAi_Trametinib	6.19	18.75	-12.56	8.055	14	12	ns

Figure EV3C	mean 1	mean 2	mean Diff	SE of Diff	n1	n2	Summary
RAP_Trametinib vs. RAP+cdc23-RNAi_Trametinib	6.19	0	6.19	13.73	14	3	ns
RAP_Trametinib vs. RAP+rpt3r-RNAi_Trametinib	6.19	4.166	2.024	9.564	14	8	ns
RAP_Trametinib vs. RAP+ter94-RNAi_Trametinib	6.19	16.67	-10.48	10.53	14	6	ns
RAP_Trametinib vs. RAP+prosalpha4-RNAi_Trametinib	6.19	0	6.19	12.23	14	4	ns
RAP_Trametinib vs. RAP+alh-RNAi_Trametinib	6.19	5	1.19	11.24	14	5	ns

RAP_Trametinib vs. RAP+punch-RNAi_Trametinib	6.19	0	6.19	10.53	14	6	ns
RAP_Trametinib vs. RAP+CG4502-RNAi_Trametinib	6.19	16.67	-10.48	11.24	14	5	ns
RAP_Trametinib vs. RAP+CG12493-RNAi_Trametinib	6.19	24	-17.81	11.24	14	5	ns
RAP_Trametinib vs. RAP+vis-RNAi_Trametinib	6.19	29.86	-23.67	8.489	14	12	ns
RAP_Trametinib vs. RAP+rpt4-RNAi_Trametinib	6.19	0	6.19	11.24	14	5	ns
RAP_Trametinib vs. RAP+rpn3-RNAi_Trametinib	6.19	0	6.19	16.31	14	2	ns

Figure EV3D	mean 1	mean 2	mean Diff	SE of Diff	n1	n2	Summary
RAP_Trametinib vs. RAP+cyp4p1-RNAi_Trametinib	6.19	14.88	-8.692	8.592	14	6	ns
RAP_Trametinib vs. RAP+hsp23-RNAi_Trametinib	6.19	0	6.19	8.592	14	6	ns
RAP_Trametinib vs. RAP+mal-a6-RNAi_Trametinib	6.19	0	6.19	9.983	14	4	ns
RAP_Trametinib vs. RAP+fng-RNAi_Trametinib	6.19	0	6.19	8.592	14	6	ns
RAP_Trametinib vs. RAP+CG30427-RNAi_Trametinib	6.19	0	6.19	11.2	14	3	ns
RAP_Trametinib vs. RAP+prosbeta3-RNAi_Trametinib	6.19	0	6.19	9.983	14	4	ns
RAP_Trametinib vs. RAP+ldh-RNAi_Trametinib	6.19	25	-18.81	9.983	14	4	ns
RAP_Trametinib vs. RAP+CG8036-RNAi_Trametinib	6.19	2.858	3.332	9.174	14	5	ns
RAP_Trametinib vs. RAP+CG32365-RNAi_Trametinib	6.19	0	6.19	9.983	14	4	ns
RAP_Trametinib vs. RAP+CG4733-RNAi_Trametinib	6.19	20.83	-14.64	9.983	14	4	ns
RAP_Trametinib vs. RAP+CG14395-RNAi_Trametinib	6.19	0	6.19	9.983	14	4	ns

Figure EV3E	mean 1	mean 2	mean Diff	SE of Diff	n1	n2	Summary
RasG12V_Trametinib vs. RasG12V+nd-pdsw-RNAi_Trametinib	93.91	92.78	1.133	21.48	13	6	ns
RasG12V_Trametinib vs. RasG12V+cda4-RNAi_Trametinib	93.91	95	-1.09	21.48	13	6	ns
RasG12V_Trametinib vs. RasG12V+muc-RNAi_Trametinib	93.91	100	-6.09	33.05	13	2	ns
RasG12V_Trametinib vs. RasG12V+CG4459-RNAi_Trametinib	93.91	73.33	20.58	22.9	13	5	ns
RasG12V_Trametinib vs. RasG12V+cox5a-RNAi_Trametinib	93.91	125	-31.09	21.48	13	6	ns
RasG12V_Trametinib vs. RasG12V+CG32564-RNAi_Trametinib	93.91	96.11	-2.202	21.48	13	6	ns
RasG12V_Trametinib vs. RasG12V+nd-b22-RNAi_Trametinib	93.91	70	23.91	21.48	13	6	ns
RasG12V_Trametinib vs. RasG12V+cox7a-RNAi_Trametinib	93.91	105.6	-11.64	21.48	13	6	ns

Figure EV3F	mean 1	mean 2	mean Diff	SE of Diff	n1	n2	Summary
RAP+dl[1]_DMSO vs. RAP+dl[1]_Trametinib	19.44	52.41	-32.96	8.572	16	18	***

RAP+dl[1]_DMSO vs. RAP_DMSO	19.44	6.669	12.78	9.13	16	14	ns
RAP+dl[1]_Trametinib vs. RAP_Trametinib	52.41	6.133	46.27	8.891	18	14	***

Figure EV3G	mean 1	mean 2	mean Diff	SE of Diff	n1	n2	Summary
RAP_DMSO vs. RAP_1µM QNZ	10.52	6.688	3.833	5.948	12	12	ns
RAP_DMSO vs. RAP_5µM QNZ	10.52	1.98	8.54	5.948	12	12	ns
RAP_Trametinib vs. RAP_Trametinib+1µM QNZ	34.51	22.63	11.88	5.948	12	12	ns
RAP_Trametinib vs. RAP_Trametinib+5µM QNZ	34.51	10.18	24.34	5.948	12	12	***

Figure EV3H	mean 1	mean 2	mean Diff	SE of Diff	n1	n2	Summary
Control_Trametinib vs. Control_Trametinib+1µM QNZ	114.1	115.2	-1.102	11.69	20	12	ns
Control_Trametinib vs. Control_Trametinib+5µM QNZ	114.1	109.7	4.394	11.69	20	12	ns

Figure EV3I	mean 1	mean 2	mean Diff	SE of Diff	n1	n2	Summary
RAP_Trametinib vs. RAP_Trametinib+1µM JSH23	12	30.6	-18.6	12.56	12	12	ns
RAP_Trametinib vs. RAP_Trametinib+5µM JSH23	12	29.87	-17.87	12.56	12	12	ns
RAP_Trametinib vs. RAP_Trametinib+10µM JSH23	12	12.74	-0.7408	12.56	12	12	ns

Figure EV4A	mean 1	mean 2	mean Diff	SE of Diff	n1	n2	Summary
RAP_Trametinib vs. RAP_Trametinib+1µM iCRT3	52.87	51.06	1.811	10.62	11	11	ns
RAP_Trametinib vs. RAP_Trametinib+5µM iCRT3	52.87	63.64	-10.76	10.89	11	10	ns
RAP_Trametinib vs. RAP_Trametinib+10µM iCRT3	52.87	41.31	11.57	10.62	11	11	ns

Figure EV4B	mean 1	mean 2	mean Diff	SE of Diff	n1	n2	Summary
RAP_Trametinib vs. RAP_Trametinib+5µM IWP-01	36.64	44.88	-8.236	9.29	28	18	ns
RAP_Trametinib vs. RAP_Trametinib+10µM IWP-01	36.64	42.36	-5.713	8.761	28	22	ns
RAP_Trametiinb vs. RAP_Trametinib+15µM IWP-01	36.64	22.18	14.47	8.877	28	21	ns

Figure EV4C	mean 1	mean 2	mean Diff	SE of Diff	n1	n2	Summary
RAP_Trametinib vs. RAP_Trametinib+1µM XAV-939	49.55	57.93	-8.378	11.74	12	12	ns
RAP_Trametinib vs. RAP_Trametinib+5µM XAV939	49.55	51.87	-2.324	11.74	12	12	ns
RAP_Trametinib vs. RAP_Trametinib+10µM XAV939	49.55	28.72	20.83	11.74	12	12	ns

Figure EV4D	mean 1	mean 2	mean Diff	SE of Diff	n1	n2	Summary
RAP_Trametinib vs. RAP_Trametinib+1µM Capmatinib	33.71	40.33	-6.622	12.25	12	12	ns
RAP_Trametinib vs. RAP_Trametinib+5µM Capmatinib	33.71	37.45	-3.738	12.25	12	12	ns
RAP_Trametinib vs. RAP_Trametinib+10µM Capmatinib	33.71	48.22	-14.51	12.25	12	12	ns

Figure EV4E		2−ΔΔCt	
Control	1	1	1
RAP	85.83	73.43	61.68
RAP_Trametinib	9.45	14.22	29.18
RAP_Trametinib+PNU	2.59	5.86	5.45

Figure EV5A	mean 1	mean 2	mean Diff	SE of Diff	n1	n2	Summary
RAP+ GFP_Trametinib+PNU vs. RAP+DNApol-eta-RNAi_Trametinib+PNU	2.857	25.6	-22.74	9.809	7	6	ns
RAP+ GFP_Trametinib+PNU vs. RAP+Irp1-RNAi_Trametinib+PNU	2.857	20.83	-17.98	11.05	7	4	ns

Figure EV5B	mean 1	mean 2	mean Diff	SE of Diff	n1	n2	Summary
RAP+GFP_Trametinib+LF3 vs. RAP+brmRNAi_Trametinib+LF3	2.983	66.83	-63.84	13.28	19	12	***
RAP+GFP_Trametinib+LF3 vs. RAP+shgRNAi_Trametinib+LF3	2.983	35.07	-32.09	12.02	19	17	*
RAP+GFP_Trametinib+LF3 vs. RAP+agoRNAi_Trametinib+LF3	2.983	66.06	-63.08	13.64	19	11	***
RAP+GFP_Trametinib+LF3 vs. RAP+rhoGAPp190RNAi_Trametinib+LF3	2.983	44.3	-41.32	13.28	19	12	*
RAP+GFP_Trametinib+LF3 vs. RAP+upf1RNAi_Trametinib+LF3	2.983	81.91	-78.92	13.28	19	12	***

Figure EV5C	mean 1	mean 2	mean Diff	SE of Diff	n1	n2	Summary
Control+GFP_DMSO vs. Control+brmRNAi_Trametinib+LF3	109.8	95.24	14.56	14.47	12	12	ns
Control+GFP_DMSO vs. Control+shgRNAi_Trametinib+LF3	109.8	91.79	18.02	14.47	12	12	ns
Control+GFP_DMSO vs. Control+agoRNAi_Trametinib+LF3	109.8	88.14	21.67	14.47	12	12	ns
Control+GFP_DMSO vs. Control+rhoGAPp190RNAi_Trametinib+LF3	109.8	104.4	5.402	14.47	12	12	ns
Control+GFP_DMSO vs. Control+upf1RNAi_Trametinib+LF3	109.8	77.26	32.55	14.47	12	12	ns

Figure EV6G	mean 1	mean 2	mean Diff	SE of Diff	n1	n2	Summary
KRASMT and β-Catenin High vs. KRASControl and β-Catenin Low	88.82	72.54	16.28	14.49	29	316	ns

KRASMT and β-Catenin High vs. KRASMT or β-Catenin High	88.82	72.23	16.59	14.74	29	223	ns

Figure EV6H	mean 1	mean 2	mean Diff	SE of Diff	n1	n2	Summary
T84-Run1							
DMSO vs. Tram	100	41.22	58.78	15.69	3	3	ns
DMSO vs. JSH-23	100	104.9	-4.867	15.81	3	3	ns
DMSO vs. Tram+JSH-23	100	27	73	16	3	3	ns
Tram vs. JSH-23	41.22	104.9	-63.65	2.573	3	3	***
Tram vs. Tram+JSH-23	41.22	27	14.22	3.59	3	3	ns
JSH-23 vs. Tram+JSH-23	104.9	27	77.86	4.053	3	3	***
T84-Run2							
DMSO vs. Tram	100	12.99	87.01	9.462	3	3	*
DMSO vs. JSH-23	100	101.1	-1.14	10.12	3	3	ns
DMSO vs. Tram+JSH-23	100	10.74	89.26	9.489	3	3	*
Tram vs. JSH-23	12.99	101.1	-88.15	3.644	3	3	**
Tram vs. Tram+JSH-23	12.99	10.74	2.25	0.9772	3	3	ns
JSH-23 vs. Tram+JSH-23	101.1	10.74	90.4	3.713	3	3	**
T84-Run3							
DMSO vs. Tram	100	29.49	70.51	6.748	3	3	**
DMSO vs. JSH-23	100	86.8	13.2	6.128	3	3	ns
DMSO vs. Tram+JSH-23	100	9.367	90.63	6.204	3	3	**
Tram vs. JSH-23	29.49	86.8	-57.31	3.521	3	3	**
Tram vs. Tram+JSH-23	29.49	9.367	20.12	3.652	3	3	*
JSH-23 vs. Tram+JSH-23	86.8	9.367	77.43	2.316	3	3	****

Figure EV6I	mean 1	mean 2	mean Diff	SE of Diff	n1	n2	Summary
SW620-Run1							
DMSO vs. Tram	100	22.04	77.96	0.615	3	3	****
DMSO vs. JSH-23	100	11.17	88.83	0.6483	3	3	****
DMSO vs. Tram+JSH-23	100	12.31	87.69	1.358	3	3	****

Tram vs. JSH-23	22.04	11.17	10.87	0.2925	3	3	****
Tram vs. Tram+JSH-23	22.04	12.31	9.727	1.228	3	3	*
JSH-23 vs. Tram+JSH-23	11.17	12.31	-1.147	1.245	3	3	ns
SW620-Run2							
DMSO vs. Tram	100	43.87	56.13	1.368	3	3	****
DMSO vs. JSH-23	100	16.43	83.57	1.388	3	3	****
DMSO vs. Tram+JSH-23	100	21.28	78.72	2.674	3	3	***
Tram vs. JSH-23	43.87	16.43	27.44	0.5009	3	3	****
Tram vs. Tram+JSH-23	43.87	21.28	22.59	2.339	3	3	*
JSH-23 vs. Tram+JSH-23	16.43	21.28	-4.849	2.351	3	3	ns
SW620-Run3							
DMSO vs. Tram	100	40.72	59.28	1.025	3	3	****
DMSO vs. JSH-23	100	13.37	86.63	1.003	3	3	****
DMSO vs. Tram+JSH-23	100	17.01	82.99	2.356	3	3	****
Tram vs. JSH-23	40.72	13.37	27.35	0.3076	3	3	****
Tram vs. Tram+JSH-23	40.72	17.01	23.71	2.153	3	3	*
JSH-23 vs. Tram+JSH-23	13.37	17.01	-3.641	2.143	3	3	ns
