# Supplementary material for: WNT signalling promotes NF-κB activation and drug resistance in KRAS-mutant colorectal cancer
Source: EMBO Rep. 2025 Nov 4;26(23):5728–55. doi: 10.1038/s44319-025-00588-1 (PMC12678608; doi:10.1038/s44319-025-00588-1)
Supplement: Supplementary file 2 — Table EV2 [file 44319_2025_588_MOESM2_ESM.pdf]

Table EV2: a summary of detailed drosophila genotypes.

Figure1:
+/+ or Y; +/+; byn-Gal4, UAS-GFP, tub-Gal80^{TS}/+ (A, B and G), +/+ or Y; UAS-Ras^{G12V}/+; byn-Gal4, UAS-GFP, tub-Gal80^{TS}/+ (A and G), +/+ or Y; UAS-Ras^{G12V}, Apc-RNAi, UAS-P53-RNAi/+; byn-Gal4, UAS-GFP, tub-Gal80^{TS}/+ (A and B), +/+ or Y; UAS-Ras^{G12V}, Apc-RNAi, UAS-P53-RNAi/UAS-GFP; byn-Gal4, UAS-GFP, tub-Gal80^{TS}/+ (H and K), +/+ or Y; UAS-Ras^{G12V}, Apc-RNAi, UAS-P53-RNAi/+; byn-Gal4, UAS-GFP, tub-Gal80^{TS}/UAS-dif-RNAi (C, J and K), +/+ or Y; UAS-Ras^{G12V}, Apc-RNAi, UAS-P53-RNAi/UAS-dl-RNAi; byn-Gal4, UAS-GFP, tub-Gal80^{TS}/+ (C, I and K), +/+ or Y; UAS-GFP/+; byn-Gal4, UAS-GFP, tub-Gal80^{TS}/+ (D and F), +/+ or Y; +/+; byn-Gal4, UAS-GFP, tub-Gal80^{TS}/UAS-dif-RNAi (D), +/+ or Y; UAS-dl-RNAi/+; byn-Gal4, UAS-GFP, tub-Gal80^{TS}/+ (D), +/+ or Y; UAS-Ras^{G12V}/UAS-GFP; byn-Gal4, UAS-GFP, tub-Gal80^{TS}/+ (E), +/+ or Y; UAS-Ras^{G12V}/UAS-cact-RNAi; byn-Gal4, UAS-GFP, tub-Gal80^{TS}/+ (E), +/+ or Y; UAS-cact-RNAi/+; byn-Gal4, UAS-GFP, tub-Gal80^{TS}/+ (F), UAS-Arm^{S10}/+ or Y; +/+; byn-Gal4, UAS-GFP, tub-Gal80^{TS}/+ (G), UAS-Arm^{S10}/+ or Y; UAS-Ras^{G12V}/+; byn-Gal4, UAS-GFP, tub-Gal80^{TS}/+ (G).

Figure2:
+/+ or Y; UAS-Ras^{G12V}, Apc-RNAi, UAS-P53-RNAi/+; byn-Gal4, UAS-GFP, tub-Gal80^{TS}/UAS-Toll1-RNAi (A and E), +/+ or Y; UAS-Ras^{G12V}, Apc-RNAi, UAS-P53-RNAi/+; byn-Gal4, UAS-GFP, tub-Gal80^{TS}/UAS-Toll9-RNAi (A and F), +/+ or Y; UAS-GFP/+; byn-Gal4, UAS-GFP, tub-Gal80^{TS}/+ (B), +/+ or Y; +/+; byn-Gal4, UAS-GFP, tub-Gal80^{TS}/UAS-Toll1-RNAi (B), +/+ or Y; +/+; byn-Gal4, UAS-GFP, tub-Gal80^{TS}/UAS-Toll9-RNAi (B), +/+ or Y; +/+; byn-Gal4, UAS-GFP, tub-Gal80^{TS}/+ (C), +/+ or Y; UAS-Ras^{G12V}, Apc-RNAi, UAS-P53-RNAi/+; byn-Gal4, UAS-GFP, tub-Gal80^{TS}/+ (D).

Figure3:
+/+ or Y; UAS-Ras^{G12V}, Apc-RNAi, UAS-P53-RNAi/UAS-GFP; byn-Gal4, UAS-GFP, tub-Gal80^{TS}/+ (A, C, G and H), +/+ or Y; UAS-Ras^{G12V}, Apc-RNAi, UAS-P53-RNAi/UAS-dl-RNAi; byn-Gal4, UAS-GFP, tub-Gal80^{TS}/+ (A, C and G), +/+ or Y; UAS-Ras^{G12V}, Apc-RNAi, UAS-P53-RNAi/UAS-blanks-RNAi; byn-Gal4, UAS-GFP, tub-Gal80^{TS}/+ (D), +/+ or Y; UAS-Ras^{G12V}, Apc-RNAi, UAS-P53-RNAi/UAS-cht4-RNAi; byn-Gal4, UAS-GFP, tub-Gal80^{TS}/+ (D and H), +/+ or Y; UAS-Ras^{G12V}, Apc-RNAi, UAS-P53-RNAi/UAS-mfs14-RNAi; byn-Gal4, UAS-GFP, tub-Gal80^{TS}/+ (D), +/+ or Y; UAS-Ras^{G12V}, Apc-RNAi, UAS-P53-RNAi/UAS-cht5-RNAi; byn-Gal4, UAS-GFP, tub-Gal80^{TS}/+ (E), +/+ or Y; UAS-GFP/+; byn-Gal4, UAS-GFP, tub-Gal80^{TS}/+ (F), +/+ or Y; UAS-blanks-RNAi/+; byn-Gal4, UAS-GFP, tub-Gal80^{TS}/+ (F), +/+ or Y; UAS-cht4-RNAi/+; byn-Gal4, UAS-GFP, tub-Gal80^{TS}/+ (F), +/+ or Y; UAS-mfs14-RNAi/+; byn-Gal4, UAS-GFP, tub-Gal80^{TS}/+ (F).

Figure4:
+/+ or Y; UAS-Ras^{G12V}, Apc-RNAi, UAS-P53-RNAi/+; byn-Gal4, UAS-GFP, tub-Gal80^{TS}/+ (A, B, C, E-G), +/+ or Y; +/+; byn-Gal4, UAS-GFP, tub-Gal80^{TS}/+ (D).

Figure5:

+/+ or Y; UAS-shg-RNAi, UAS-put-RNAi, UAS-p38a-RNAi, UAS-ft-RNAi, UAS-brm-RNAi, UAS-ago-RNAi, UAS-apc-RNAi, UAS-p53-RNAi, UAS-Ras^{G12V}/+; byn-Gal4, UAS-GFP, tub-Gal80^{TS}/UAS-ago-RNAi, UAS-apc-RNAi (A, CPCT006), *+/+ or Y; UAS-pten-RNAi, UAS-pten-RNAi, UAS-apc-RNAi, UAS-apc-RNAi, UAS-smox-RNAi, UAS-smox-RNAi, UAS-p53-RNAi, UAS-p53-RNAi, UAS-Ras^{G12V}/+; byn-Gal4, UAS-GFP, tub-Gal80^{TS}/UAS-upf1--RNAi, UAS-nej-RNAi, UASrhoGAPp190-RNAi, UAS-tefu-RNAi, UAS-apc-RNAi,UAS-smox-RNAi, UAS-pten-RNAi, UAS-p53-RNAi* (B, CPCT018), *+/+ or Y; UAS-lrp1-RNAi, UAS-dnapol-eta-RNAi, UAS-rad51c-RNAi, UAS-pc-RNAi, UAS-CG13344-RNAi, UAS-nos-RNAi, UAS-smox-RNAi, UAS-p53-RNAi, pvr/+; byn-Gal4, UAS-GFP, tub-Gal80^{TS}/ UAS-Ras^{G12V}, UAS-apc-RNAi, UAS-apc-RNAi, UAS-apc-RNAi, UAS-apc-RNAi, UAS-smox-RNAi, UAS-smox-RNAi, UAS-p53-RNAi, UAS-p53-RNAi* (C, CPCT045). *+/+ or Y; UAS-ird1-RNAi, UAS-wdb-RNAi, UAS-scat-RNAi, UAS-debcl-RNAi, UAS-smox-RNAi, UAS-smox-RNAi, UAS-pten-RNAi, UAS-pten-RNAi, pvr, chico/+; byn-Gal4, UAS-GFP, tub-Gal80^{TS}/ UAS-Ras^{G12V}, UAS-apc-RNAi, UAS-apc-RNAi, UAS-apc-RNAi, UAS-apc-RNAi, UAS-p53-RNAi, UAS-p53-RNAi, UAS-p53-RNAi, UAS-p53-RNAi* (D, CPCT050), *+/+ or Y; UAS-med-RNAi, UAS-med-RNAi, UAS-apc-RNAi, UAS-apc-RNAi, UAS-smox-RNAi, UAS-smox-RNAi, UAS-p53-RNAi, UAS-p53-RNAi, chico, UAS-Ras^{G12V}/+; byn-Gal4, UAS-GFP, tub-Gal80^{TS}/UAS-mus81-RNAi, UAS-pi4kIIIα-RNAi, UAS-CG4238-RNAi, UAS-fur2-RNAi, UAS-trr-RNAi, UAS-med-RNAi, UAS-p53-RNAi, UAS-apc-RNAi* (E, CPCT029), *+/+ or Y; UAS-ago-RNAi, UAS-wts-RNAi, UAS-CG7742-RNAi, UAS-Atg2-RNAi, UAS-Ras^{G12V}, Apc-RNAi, UAS-P53-RNAi/UAS-GFP; byn-Gal4, UAS-GFP, tub-Gal80^{TS}/+* (RAPp1, F), *+/+ or Y; UAS-vrp1-RNAi, UAS-ry-RNAi, UAS-khc-73-RNAi, UAS-Ras^{G12V}, Apc-RNAi, UAS-P53-RNAi/UAS-GFP; byn-Gal4, UAS-GFP, tub-Gal80^{TS}/+* (RAPp2, F).

Figure6:

+/+ or Y; UAS-Ras^{G12V}, Apc-RNAi, UAS-P53-RNAi/UAS-GFP; byn-Gal4, UAS-GFP, tub-Gal80^{TS}/+ (A, C, E and F), *+/+ or Y; UAS-Ras^{G12V}, Apc-RNAi, UAS-P53-RNAi/+; byn-Gal4, UAS-GFP, tub-Gal80^{TS}/UAS-brm-RNAi* (A, C, E and F), *+/+ or Y; UAS-Ras^{G12V}, Apc-RNAi, UAS-P53-RNAi/+; byn-Gal4, UAS-GFP, tub-Gal80^{TS}/UAS-ago-RNAi* (A, C, E and F), *+/+ or Y; UAS-Ras^{G12V}, Apc-RNAi, UAS-P53-RNAi/UAS-shg-RNAi; byn-Gal4, UAS-GFP, tub-Gal80^{TS}/+* (A, C, E and F), *+/+ or Y; UAS-Ras^{G12V}, Apc-RNAi, UAS-P53-RNAi/UAS-upf1-RNAi; byn-Gal4, UAS-GFP, tub-Gal80^{TS}/+* (A, C, E and F), *+/+ or Y; UAS-Ras^{G12V}, Apc-RNAi, UAS-P53-RNAi/UAS-rhoGAPp190-RNAi; byn-Gal4, UAS-GFP, tub-Gal80^{TS}/+* (A, C, E and F), *+/+ or Y; UAS-Ras^{G12V}, Apc-RNAi, UAS-P53-RNAi/+; byn-Gal4, UAS-GFP, tub-Gal80^{TS}/UAS-CG13344-RNAi* (A), *+/+ or Y; UAS-Ras^{G12V}, Apc-RNAi, UAS-P53-RNAi/+; byn-Gal4, UAS-GFP, tub-Gal80^{TS}/UAS-p38a-RNAi* (A), *+/+ or Y; UAS-Ras^{G12V}, Apc-RNAi, UAS-P53-RNAi/+; byn-Gal4, UAS-GFP, tub-Gal80^{TS}/UAS-ft-RNAi* (A), *+/+ or Y; UAS-Ras^{G12V}, Apc-RNAi, UAS-P53-RNAi/UAS-put-RNAi; byn-Gal4, UAS-GFP, tub-Gal80^{TS}/+* (A), *+/+ or Y; +/UAS-GFP; byn-Gal4, UAS-GFP, tub-Gal80^{TS}/+* (B), *+/+ or Y; +/+; byn-Gal4, UAS-GFP, tub-Gal80^{TS}/UAS-brm-RNAi* (B), *+/+ or Y; +/+; byn-Gal4, UAS-GFP, tub-Gal80^{TS}/UAS-ago-RNAi* (B), *+/+ or Y; +/UAS-shg-RNAi; byn-Gal4, UAS-GFP, tub-Gal80^{TS}/+* (B), *+/+ or Y; +/UAS-upf1-RNAi; byn-Gal4, UAS-GFP, tub-Gal80^{TS}/+* (B), *+/+ or Y; +/UAS-rhoGAPp190-RNAi; byn-Gal4, UAS-GFP, tub-Gal80^{TS}/+* (B), *+/+ or Y; UAS-ago-RNAi, UAS-wts-RNAi, UAS-CG7742-RNAi, UAS-Atg2-RNAi, UAS-Ras^{G12V}, Apc-RNAi,*

UAS-P53-RNAi/UAS-GFP; byn-Gal4, UAS-GFP, tub-Gal80^{TS}/+ (RAPp1, G), +/+ or Y; UAS-ago-RNAi, UAS-wts-RNAi, UAS-CG7742-RNAi, UAS-Atg2-RNAi, UAS-Ras^{G12V}, Apc-RNAi, UAS-P53-RNAi/+; byn-Gal4, UAS-GFP, tub-Gal80^{TS}/UAS-brm-RNAi (RAPp1, G), +/+ or Y; UAS-ago-RNAi, UAS-wts-RNAi, UAS-CG7742-RNAi, UAS-Atg2-RNAi, UAS-Ras^{G12V}, Apc-RNAi, UAS-P53-RNAi/+; byn-Gal4, UAS-GFP, tub-Gal80^{TS}/UAS-ago-RNAi (RAPp1, G), +/+ or Y; UAS-ago-RNAi, UAS-wts-RNAi, UAS-CG7742-RNAi, UAS-Atg2-RNAi, UAS-Ras^{G12V}, Apc-RNAi, UAS-P53-RNAi/UAS-shg-RNAi; byn-Gal4, UAS-GFP, tub-Gal80^{TS}/+ (RAPp1, G), +/+ or Y; UAS-ago-RNAi, UAS-wts-RNAi, UAS-CG7742-RNAi, UAS-Atg2-RNAi, UAS-Ras^{G12V}, Apc-RNAi, UAS-P53-RNAi/UAS-upf1-RNAi; byn-Gal4, UAS-GFP, tub-Gal80^{TS}/+ (RAPp1, G), +/+ or Y; UAS-ago-RNAi, UAS-wts-RNAi, UAS-CG7742-RNAi, UAS-Atg2-RNAi, UAS-Ras^{G12V}, Apc-RNAi, UAS-P53-RNAi/UAS-rhoGAPp190-RNAi; byn-Gal4, UAS-GFP, tub-Gal80^{TS}/+ (RAPp1, G), +/+ or Y; UAS-vrp1-RNAi, UAS-ry-RNAi, UAS-khc-73-RNAi, UAS-Ras^{G12V}, Apc-RNAi, UAS-P53-RNAi/UAS-GFP; byn-Gal4, UAS-GFP, tub-Gal80^{TS}/+ (RAPp2, H), +/+ or Y; UAS-vrp1-RNAi, UAS-ry-RNAi, UAS-khc-73-RNAi, UAS-Ras^{G12V}, Apc-RNAi, UAS-P53-RNAi/+; byn-Gal4, UAS-GFP, tub-Gal80^{TS}/UAS-brm-RNAi (RAPp2, H), +/+ or Y; UAS-vrp1-RNAi, UAS-ry-RNAi, UAS-khc-73-RNAi, UAS-Ras^{G12V}, Apc-RNAi, UAS-P53-RNAi/+; byn-Gal4, UAS-GFP, tub-Gal80^{TS}/UAS-ago-RNAi (RAPp2, H), +/+ or Y; UAS-vrp1-RNAi, UAS-ry-RNAi, UAS-khc-73-RNAi, UAS-Ras^{G12V}, Apc-RNAi, UAS-P53-RNAi/UAS-shg-RNAi; byn-Gal4, UAS-GFP, tub-Gal80^{TS}/+ (RAPp2, H), +/+ or Y; UAS-vrp1-RNAi, UAS-ry-RNAi, UAS-khc-73-RNAi, UAS-Ras^{G12V}, Apc-RNAi, UAS-P53-RNAi/UAS-upf1-RNAi; byn-Gal4, UAS-GFP, tub-Gal80^{TS}/+ (RAPp2, H), +/+ or Y; UAS-vrp1-RNAi, UAS-ry-RNAi, UAS-khc-73-RNAi, UAS-Ras^{G12V}, Apc-RNAi, UAS-P53-RNAi/UAS-rhoGAPp190-RNAi; byn-Gal4, UAS-GFP, tub-Gal80^{TS}/+ (RAPp2, H).

Figure EV1:

+/+ or Y; +/+; byn-Gal4, UAS-GFP, tub-Gal80^{TS}/+ (A-E), +/+ or Y; UAS-Ras^{G12V}/+; byn-Gal4, UAS-GFP, tub-Gal80^{TS}/+ (B, C, D, F and G), UAS-Arm^{S10}/+ or Y; +/+; byn-Gal4, UAS-GFP, tub-Gal80^{TS}/+ (C and H), UAS-Arm^{S10}/+ or Y; UAS-Ras^{G12V}/+; byn-Gal4, UAS-GFP, tub-Gal80^{TS}/+ (C and I), +/+ or Y; UAS-Ras^{G12V}, Apc-RNAi, UAS-P53-RNAi/+; byn-Gal4, UAS-GFP, tub-Gal80^{TS}/+ (D and K). +/+ or Y; UAS-Ras^{G12V}, UAS-P53-RNAi/+; byn-Gal4, UAS-GFP, tub-Gal80^{TS}/+ (D and J).

Figure EV2:

+/+ or Y; UAS-Ras^{G12V}, Apc-RNAi, UAS-P53-RNAi/+; byn-Gal4, UAS-GFP, tub-Gal80^{TS}/UAS-Toll3-RNAi (A), +/+ or Y; UAS-Ras^{G12V}, Apc-RNAi, UAS-P53-RNAi/+; byn-Gal4, UAS-GFP, tub-Gal80^{TS}/UAS-Toll4-RNAi (A), +/+ or Y; UAS-Ras^{G12V}, Apc-RNAi, UAS-P53-RNAi/+; byn-Gal4, UAS-GFP, tub-Gal80^{TS}/UAS-Toll5-RNAi (A), +/+ or Y; UAS-Ras^{G12V}, Apc-RNAi, UAS-P53-RNAi/+; byn-Gal4, UAS-GFP, tub-Gal80^{TS}/UAS-Toll6-RNAi (A), +/+ or Y; UAS-Ras^{G12V}, Apc-RNAi, UAS-P53-RNAi/+; byn-Gal4, UAS-GFP, tub-Gal80^{TS}/UAS-Toll7-RNAi (A), +/+ or Y; UAS-Ras^{G12V}, Apc-RNAi, UAS-P53-RNAi/+; byn-Gal4, UAS-GFP, tub-Gal80^{TS}/UAS-Toll8-RNAi (A), +/+ or Y; UAS-Ras^{G12V}, Apc-RNAi, UAS-P53-RNAi/UAS-GFP; byn-Gal4, UAS-GFP, tub-Gal80^{TS}/+ (D), +/+ or Y; UAS-Ras^{G12V}, Apc-RNAi, UAS-P53-RNAi/+; byn-Gal4, UAS-GFP, tub-Gal80^{TS}/UAS-Toll1-RNAi (B and D), +/+ or Y; UAS-Ras^{G12V}, Apc-RNAi, UAS-P53-RNAi/+; byn-Gal4, UAS-GFP, tub-

Gal80^{TS}/UAS-Toll9-RNAi (C and D).

Figure EV3:

+/+ or Y; UAS-Ras^{G12V}, Apc-RNAi, UAS-P53-RNAi/+; byn-Gal4, UAS-GFP, tub-Gal80^{TS}/UAS-rel-RNAi (A), *+/+ or Y; UAS-Ras^{G12V}, Apc-RNAi, UAS-P53-RNAi/UAS-domeHMJ21208-RNAi; byn-Gal4, UAS-GFP, tub-Gal80^{TS}/+* (A), *+/+ or Y; UAS-Ras^{G12V}, Apc-RNAi, UAS-P53-RNAi/+; byn-Gal4, UAS-GFP, tub-Gal80^{TS}/UAS-domeHMS01293-RNAi* (A), *+/+ or Y; UAS-Ras^{G12V}, Apc-RNAi, UAS-P53-RNAi/UAS-bsk-RNAi; byn-Gal4, UAS-GFP, tub-Gal80^{TS}/+* (A), *+/+ or Y; UAS-Ras^{G12V}, Apc-RNAi, UAS-P53-RNAi/UAS-GFP; byn-Gal4, UAS-GFP, tub-Gal80^{TS}/+* (B), *+/+ or Y; UAS-Ras^{G12V}, Apc-RNAi, UAS-P53-RNAi/+; byn-Gal4, UAS-GFP, tub-Gal80^{TS}/UAS-CG9360-RNAi* (B), *+/+ or Y; UAS-Ras^{G12V}, Apc-RNAi, UAS-P53-RNAi/+; byn-Gal4, UAS-GFP, tub-Gal80^{TS}/UAS-ref(2)p-RNAi* (B), *+/+ or Y; UAS-Ras^{G12V}, Apc-RNAi, UAS-P53-RNAi/UAS-CG32302-RNAi; byn-Gal4, UAS-GFP, tub-Gal80^{TS}/+* (B), *+/+ or Y; UAS-Ras^{G12V}, Apc-RNAi, UAS-P53-RNAi/UAS-CG17104-RNAi; byn-Gal4, UAS-GFP, tub-Gal80^{TS}/+* (B), *+/+ or Y; UAS-Ras^{G12V}, Apc-RNAi, UAS-P53-RNAi/UAS-mec2-RNAi; byn-Gal4, UAS-GFP, tub-Gal80^{TS}/+* (B), *+/+ or Y; UAS-Ras^{G12V}, Apc-RNAi, UAS-P53-RNAi/UAS-CG1698-RNAi; byn-Gal4, UAS-GFP, tub-Gal80^{TS}/+* (B), *+/+ or Y; UAS-Ras^{G12V}, Apc-RNAi, UAS-P53-RNAi/UAS-CG15739-RNAi; byn-Gal4, UAS-GFP, tub-Gal80^{TS}/+* (B), *+/+ or Y; UAS-Ras^{G12V}, Apc-RNAi, UAS-P53-RNAi/UAS-ag5r-RNAi; byn-Gal4, UAS-GFP, tub-Gal80^{TS}/+* (B), *+/+ or Y; UAS-Ras^{G12V}, Apc-RNAi, UAS-P53-RNAi/+; byn-Gal4, UAS-GFP, tub-Gal80^{TS}/UAS-arc1-RNAi* (B), *+/+ or Y; UAS-Ras^{G12V}, Apc-RNAi, UAS-P53-RNAi/UAS-CG2065-RNAi; byn-Gal4, UAS-GFP, tub-Gal80^{TS}/+* (B), *+/+ or Y; UAS-Ras^{G12V}, Apc-RNAi, UAS-P53-RNAi/UAS-CG10182-RNAi; byn-Gal4, UAS-GFP, tub-Gal80^{TS}/+* (B), *+/+ or Y; UAS-Ras^{G12V}, Apc-RNAi, UAS-P53-RNAi/UAS-CG18473-RNAi; byn-Gal4, UAS-GFP, tub-Gal80^{TS}/+* (B), *+/+ or Y; UAS-Ras^{G12V}, Apc-RNAi, UAS-P53-RNAi/UAS-GFP; byn-Gal4, UAS-GFP, tub-Gal80^{TS}/+* (C), *+/+ or Y; UAS-Ras^{G12V}, Apc-RNAi, UAS-P53-RNAi/UAS-cdc23-RNAi; byn-Gal4, UAS-GFP, tub-Gal80^{TS}/+* (C), *+/+ or Y; UAS-Ras^{G12V}, Apc-RNAi, UAS-P53-RNAi/UAS-rpt3r-RNAi; byn-Gal4, UAS-GFP, tub-Gal80^{TS}/+* (C), *+/+ or Y; UAS-Ras^{G12V}, Apc-RNAi, UAS-P53-RNAi/+; byn-Gal4, UAS-GFP, tub-Gal80^{TS}/UAS-ter94-RNAi* (C), *+/+ or Y; UAS-Ras^{G12V}, Apc-RNAi, UAS-P53-RNAi/UAS-prosalpha-RNAi; byn-Gal4, UAS-GFP, tub-Gal80^{TS}/+* (C), *+/+ or Y; UAS-Ras^{G12V}, Apc-RNAi, UAS-P53-RNAi/+; byn-Gal4, UAS-GFP, tub-Gal80^{TS}/UAS-alh-RNAi* (C), *+/+ or Y; UAS-Ras^{G12V}, Apc-RNAi, UAS-P53-RNAi/UAS-punch-RNAi; byn-Gal4, UAS-GFP, tub-Gal80^{TS}/+* (C), *+/+ or Y; UAS-Ras^{G12V}, Apc-RNAi, UAS-P53-RNAi/+; byn-Gal4, UAS-GFP, tub-Gal80^{TS}/UAS-CG4502-RNAi* (C), *+/+ or Y; UAS-Ras^{G12V}, Apc-RNAi, UAS-P53-RNAi/+; byn-Gal4, UAS-GFP, tub-Gal80^{TS}/UAS-CG12493-RNAi* (C), *+/+ or Y; UAS-Ras^{G12V}, Apc-RNAi, UAS-P53-RNAi/+; byn-Gal4, UAS-GFP, tub-Gal80^{TS}/UAS-vis-RNAi* (C), *+/+ or Y; UAS-Ras^{G12V}, Apc-RNAi, UAS-P53-RNAi/+; byn-Gal4, UAS-GFP, tub-Gal80^{TS}/UAS-rpt4-RNAi* (C), *+/+ or Y; UAS-Ras^{G12V}, Apc-RNAi, UAS-P53-RNAi/+; byn-Gal4, UAS-GFP, tub-Gal80^{TS}/UAS-rpn3-RNAi* (C), *+/+ or Y; UAS-Ras^{G12V}, Apc-RNAi, UAS-P53-RNAi/UAS-GFP; byn-Gal4, UAS-GFP, tub-Gal80^{TS}/+* (D), *+/+ or Y; UAS-Ras^{G12V}, Apc-RNAi, UAS-P53-RNAi/UAS-cyp4p1-RNAi; byn-Gal4, UAS-GFP, tub-Gal80^{TS}/+* (D), *+/+ or Y; UAS-Ras^{G12V}, Apc-RNAi, UAS-P53-RNAi/UAS-hsp23-RNAi; byn-Gal4, UAS-GFP, tub-Gal80^{TS}/+* (D), *+/+ or Y; UAS-Ras^{G12V}, Apc-RNAi, UAS-*

P53-RNAi/UAS-mal-a6-RNAi; byn-Gal4, UAS-GFP, tub-Gal80^{TS}/+ (D), *+/+ or Y; UAS-Ras^{G12V}, Apc-RNAi, UAS-P53-RNAi/+; byn-Gal4, UAS-GFP, tub-Gal80^{TS}/UAS-fng-RNAi* (D), *+/+ or Y; UAS-Ras^{G12V}, Apc-RNAi, UAS-P53-RNAi/UAS-CG30427-RNAi; byn-Gal4, UAS-GFP, tub-Gal80^{TS}/+* (D), *+/+ or Y; UAS-Ras^{G12V}, Apc-RNAi, UAS-P53-RNAi/+; byn-Gal4, UAS-GFP, tub-Gal80^{TS}/UAS-prosbeta3-RNAi* (D), *+/+ or Y; UAS-Ras^{G12V}, Apc-RNAi, UAS-P53-RNAi/UAS-idh-RNAi; byn-Gal4, UAS-GFP, tub-Gal80^{TS}/+* (D), *+/+ or Y; UAS-Ras^{G12V}, Apc-RNAi, UAS-P53-RNAi/UAS-CG8036-RNAi; byn-Gal4, UAS-GFP, tub-Gal80^{TS}/+* (D), *+/+ or Y; UAS-Ras^{G12V}, Apc-RNAi, UAS-P53-RNAi/UAS-CG32365-RNAi; byn-Gal4, UAS-GFP, tub-Gal80^{TS}/+* (D), *+/+ or Y; UAS-Ras^{G12V}, Apc-RNAi, UAS-P53-RNAi/UAS-CG4733-RNAi; byn-Gal4, UAS-GFP, tub-Gal80^{TS}/+* (D), *+/+ or Y; UAS-Ras^{G12V}, Apc-RNAi, UAS-P53-RNAi/UAS-CG14395-RNAi; byn-Gal4, UAS-GFP, tub-Gal80^{TS}/+* (D), *+/+ or Y; UAS-Ras^{G12V}/UAS-GFP; byn-Gal4, UAS-GFP, tub-Gal80^{TS}/+* (E), *+/+ or Y; UAS-Ras^{G12V}/+; byn-Gal4, UAS-GFP, tub-Gal80^{TS}/UAS-nd-pdsw-RNAi* (E), *+/+ or Y; UAS-Ras^{G12V}/UAS-cda4-RNAi; byn-Gal4, UAS-GFP, tub-Gal80^{TS}/+* (E), *+/+ or Y; UAS-Ras^{G12V}/+; byn-Gal4, UAS-GFP, tub-Gal80^{TS}/UAS-muc-RNAi* (E), *+/+ or Y; UAS-Ras^{G12V}/UAS-CG4459-RNAi; byn-Gal4, UAS-GFP, tub-Gal80^{TS}/+* (E), *+/+ or Y; UAS-Ras^{G12V}/UAS-cox5a-RNAi; byn-Gal4, UAS-GFP, tub-Gal80^{TS}/+* (E), *+/+ or Y; UAS-Ras^{G12V}/UAS-CG32564-RNAi; byn-Gal4, UAS-GFP, tub-Gal80^{TS}/+* (E), *+/+ or Y; UAS-Ras^{G12V}/UAS-nd-b22-RNAi; byn-Gal4, UAS-GFP, tub-Gal80^{TS}/+* (E), *+/+ or Y; UAS-Ras^{G12V}/UAS-cox7a-RNAi; byn-Gal4, UAS-GFP, tub-Gal80^{TS}/+* (E), *+/+ or Y; UAS-Ras^{G12V}, Apc-RNAi, UAS-P53-RNAi/+; byn-Gal4, UAS-GFP, tub-Gal80^{TS}/+* (F, G and I), *+/+ or Y; UAS-Ras^{G12V}, Apc-RNAi, UAS-P53-RNAi/dl[1]; byn-Gal4, UAS-GFP, tub-Gal80^{TS}/+* (F), *+/+ or Y; +/+; byn-Gal4, UAS-GFP, tub-Gal80^{TS}/+* (H).

Figure EV4:
+/+ or Y; UAS-Ras^{G12V}, Apc-RNAi, UAS-P53-RNAi/+; byn-Gal4, UAS-GFP, tub-Gal80^{TS}/+ (A-E), *+/+ or Y; +/+; byn-Gal4, UAS-GFP, tub-Gal80^{TS}/+* (E).

Figure EV5:
+/+ or Y; UAS-Ras^{G12V}, Apc-RNAi, UAS-P53-RNAi/UAS-GFP; byn-Gal4, UAS-GFP, tub-Gal80^{TS}/+ (A and B), *+/+ or Y; UAS-Ras^{G12V}, Apc-RNAi, UAS-P53-RNAi/+; byn-Gal4, UAS-GFP, tub-Gal80^{TS}/UAS-DNApol-eta-RNAi* (A), *+/+ or Y; UAS-Ras^{G12V}, Apc-RNAi, UAS-P53-RNAi/+; byn-Gal4, UAS-GFP, tub-Gal80^{TS}/UAS-lrp1-RNAi* (A), *+/+ or Y; UAS-Ras^{G12V}, Apc-RNAi, UAS-P53-RNAi/UAS-tefu-RNAi; byn-Gal4, UAS-GFP, tub-Gal80^{TS}/+* (A), *+/+ or Y; UAS-Ras^{G12V}, Apc-RNAi, UAS-P53-RNAi/+; byn-Gal4, UAS-GFP, tub-Gal80^{TS}/UAS-nej-RNAi* (A), *+/+ or Y; UAS-Ras^{G12V}, Apc-RNAi, UAS-P53-RNAi/+; byn-Gal4, UAS-GFP, tub-Gal80^{TS}/UAS-nos-RNAi* (A), *+/+ or Y; UAS-Ras^{G12V}, Apc-RNAi, UAS-P53-RNAi/UAS-pc-RNAi; byn-Gal4, UAS-GFP, tub-Gal80^{TS}/+* (A), *+/+ or Y; UAS-Ras^{G12V}, Apc-RNAi, UAS-P53-RNAi/UAS-rad51C-RNAi; byn-Gal4, UAS-GFP, tub-Gal80^{TS}/+* (A), *+/+ or Y; UAS-Ras^{G12V}, Apc-RNAi, UAS-P53-RNAi/UAS-GFP; byn-Gal4, UAS-GFP, tub-Gal80^{TS}/+* (B), *+/+ or Y; UAS-Ras^{G12V}, Apc-RNAi, UAS-P53-RNAi/+; byn-Gal4, UAS-GFP, tub-Gal80^{TS}/UAS-brm-RNAi* (B), *+/+ or Y; UAS-Ras^{G12V}, Apc-RNAi, UAS-P53-RNAi/+; byn-Gal4, UAS-GFP, tub-Gal80^{TS}/UAS-ago-RNAi* (B), *+/+ or Y; UAS-Ras^{G12V}, Apc-RNAi, UAS-P53-RNAi/UAS-shg-RNAi; byn-Gal4, UAS-GFP, tub-Gal80^{TS}/+* (B), *+/+ or Y; UAS-Ras^{G12V},*

Apc-RNAi, UAS-P53-RNAi/UAS-upf1-RNAi; byn-Gal4, UAS-GFP, tub-Gal80^{TS}/+ (B), *+/+ or Y; UAS-Ras^{G12V}, Apc-RNAi, UAS-P53-RNAi/UAS-rhoGAPp190-RNAi; byn-Gal4, UAS-GFP, tub-Gal80^{TS}/+* (B), *+/+ or Y; +/UAS-GFP; byn-Gal4, UAS-GFP, tub-Gal80^{TS}/+* (C), *+/+ or Y; +/+; byn-Gal4, UAS-GFP, tub-Gal80^{TS}/UAS-brm-RNAi* (C), *+/+ or Y; +/+; byn-Gal4, UAS-GFP, tub-Gal80^{TS}/UAS-ago-RNAi* (C), *+/+ or Y; +/UAS-shg-RNAi; byn-Gal4, UAS-GFP, tub-Gal80^{TS}/+* (C), *+/+ or Y; +/UAS-upf1-RNAi; byn-Gal4, UAS-GFP, tub-Gal80^{TS}/+* (C), *+/+ or Y; +/UAS-rhoGAPp190-RNAi; byn-Gal4, UAS-GFP, tub-Gal80^{TS}/+* (C).
