# Supplementary figures and images for: WNT signalling promotes NF-κB activation and drug resistance in KRAS-mutant colorectal cancer
Source: EMBO Rep. 2025 Nov 4;26(23):5728–55. doi: 10.1038/s44319-025-00588-1 (PMC12678608; doi:10.1038/s44319-025-00588-1)

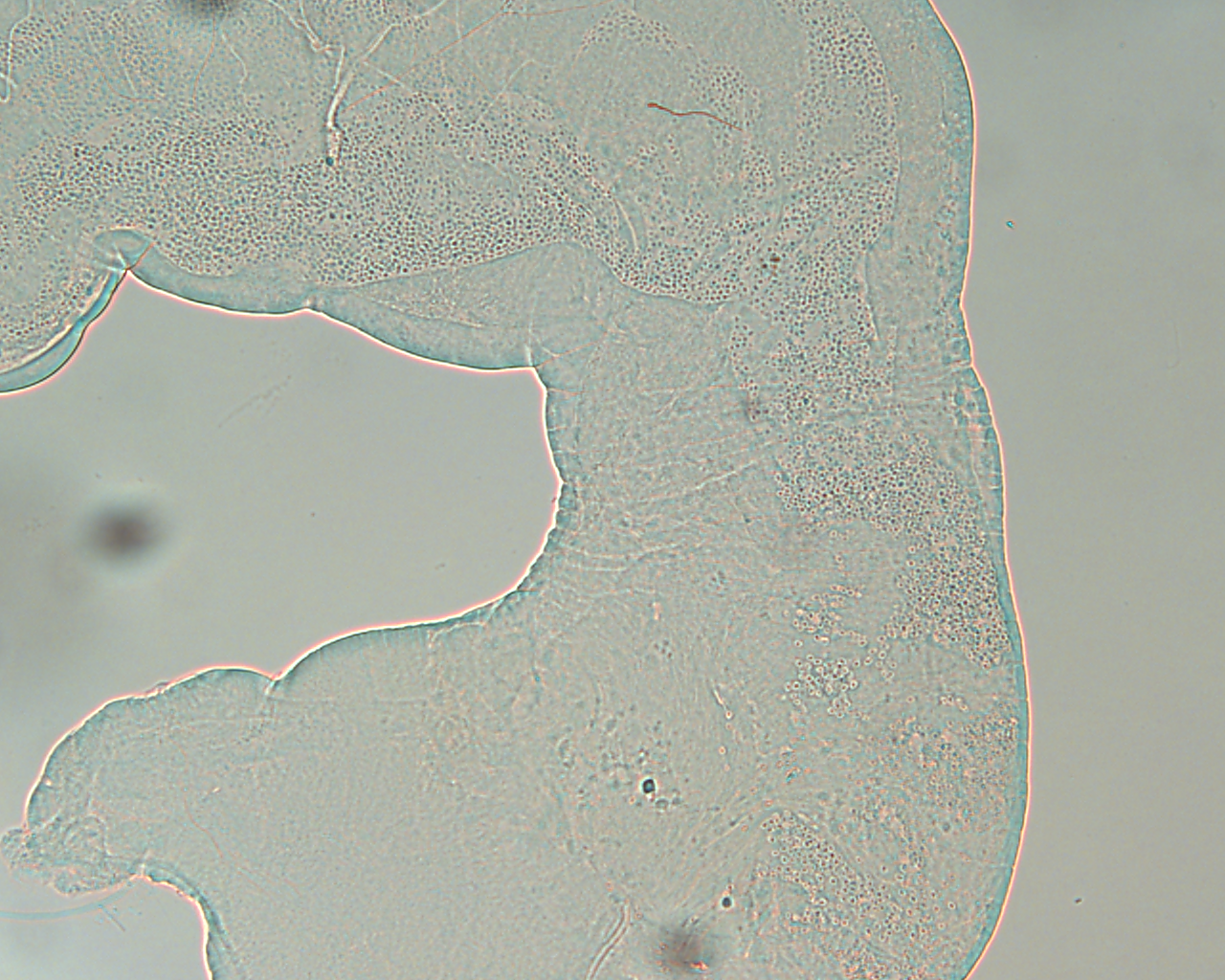

Supplement: Supplementary file 5 — Source data Fig. 1 [file 44319_2025_588_MOESM5_ESM.zip › Figure 1/Figure 1H Image data/Figure 1H.tiff]

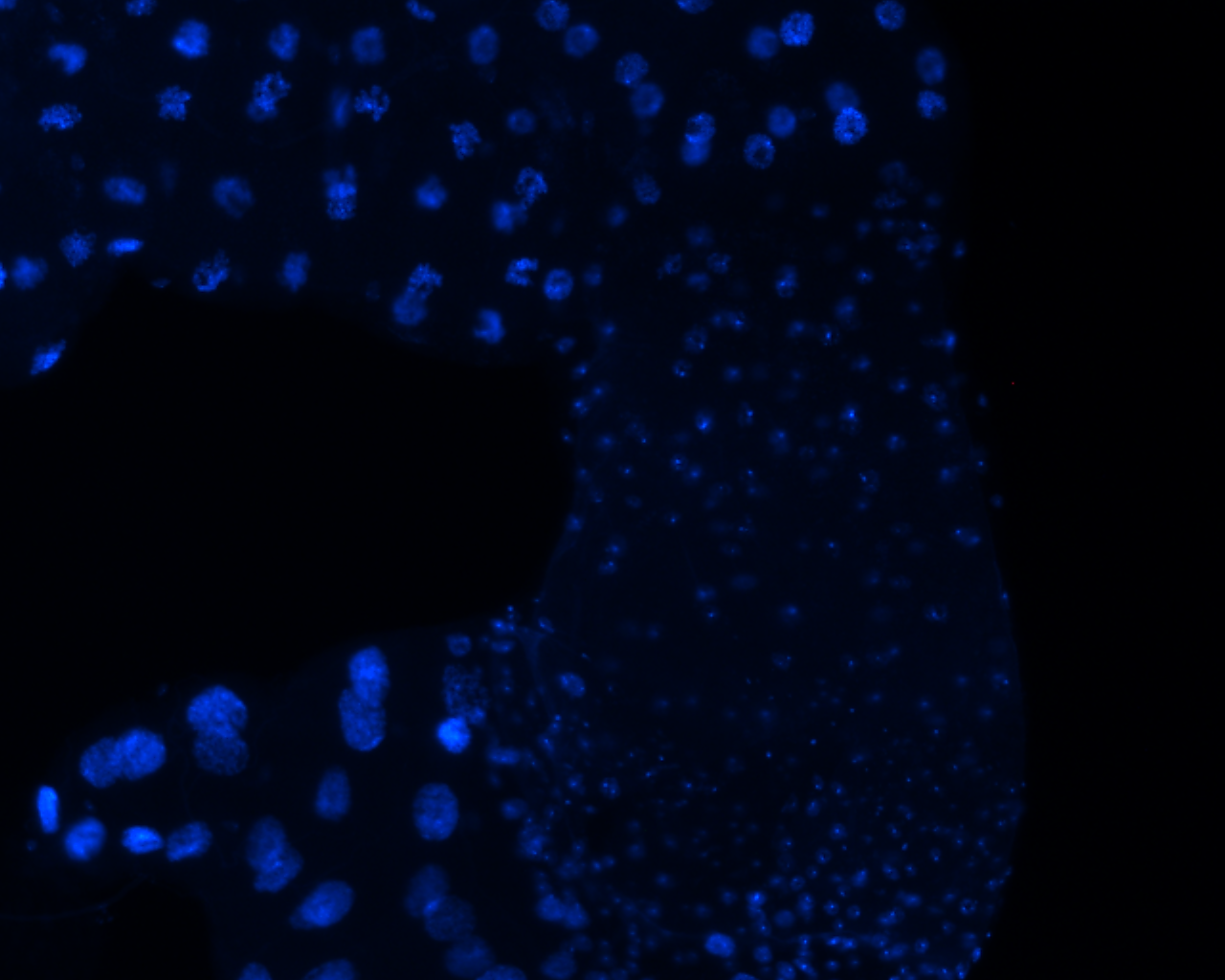

Supplement: Supplementary file 5 — Source data Fig. 1 [file 44319_2025_588_MOESM5_ESM.zip › Figure 1/Figure 1H Image data/Figure 1H'.tiff]

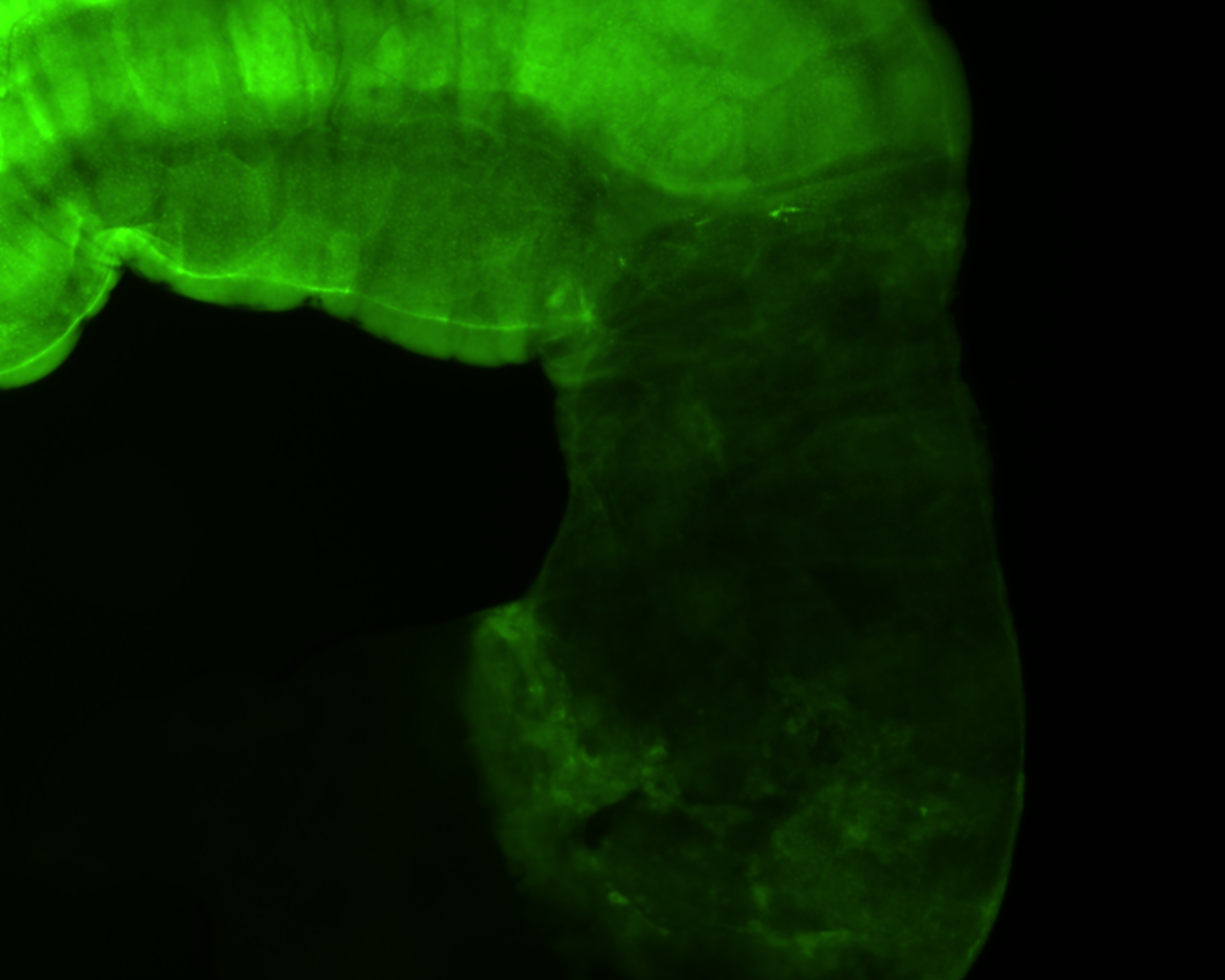

Supplement: Supplementary file 5 — Source data Fig. 1 [file 44319_2025_588_MOESM5_ESM.zip › Figure 1/Figure 1H Image data/Figure 1H''.tiff]

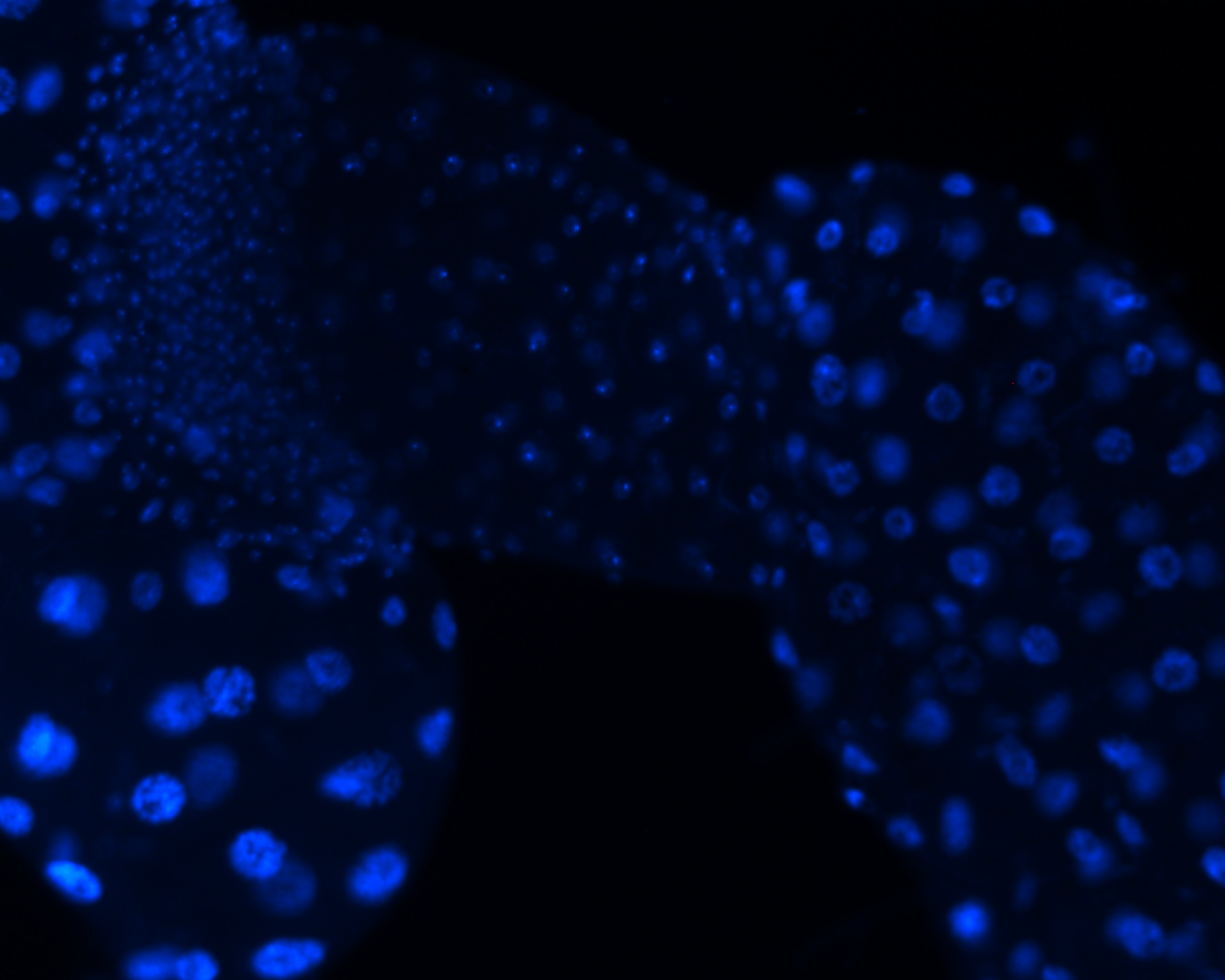

Supplement: Supplementary file 5 — Source data Fig. 1 [file 44319_2025_588_MOESM5_ESM.zip › Figure 1/Figure 1J Image data/Figure 1J'.tiff]

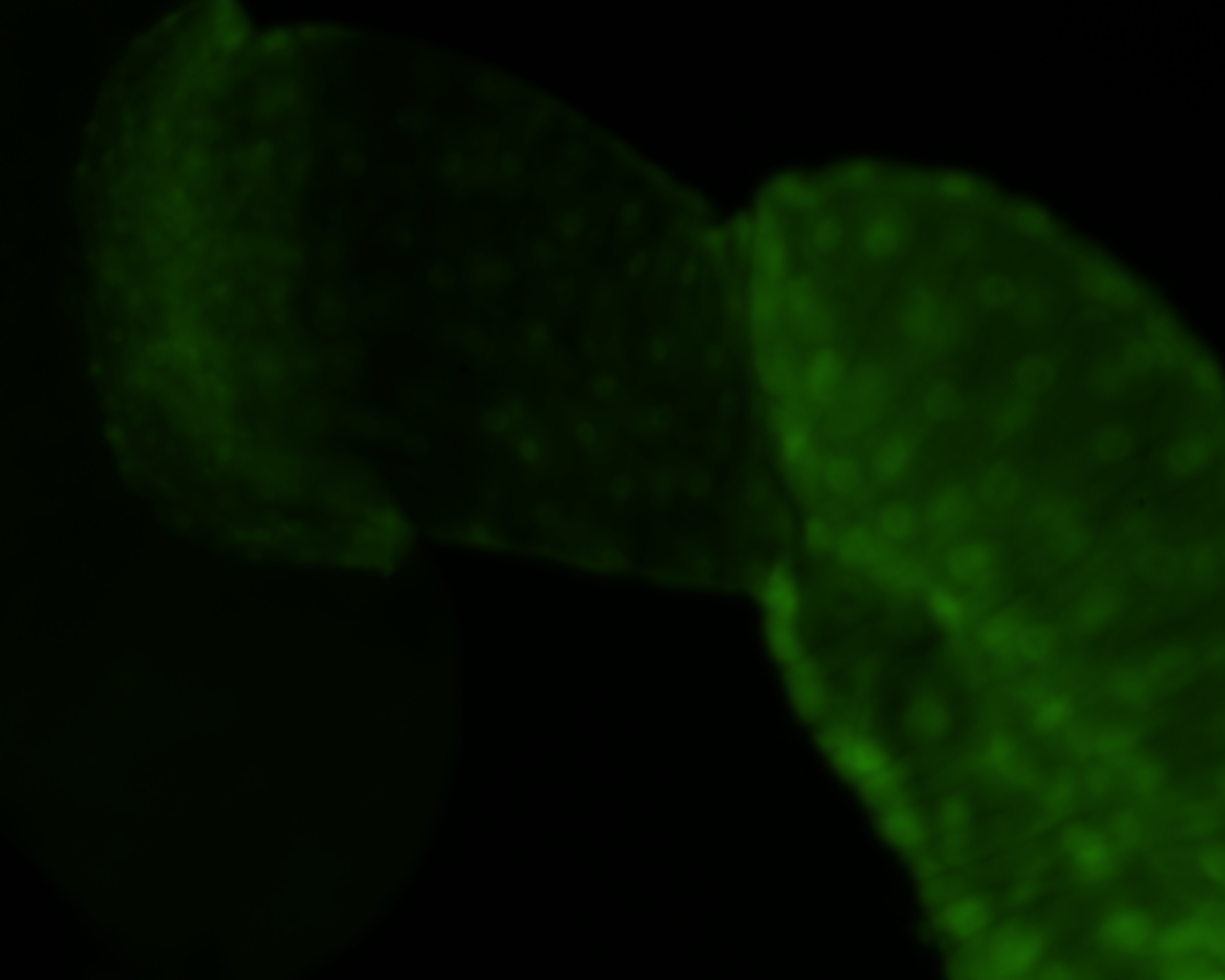

Supplement: Supplementary file 5 — Source data Fig. 1 [file 44319_2025_588_MOESM5_ESM.zip › Figure 1/Figure 1J Image data/Figure 1J''.tiff]

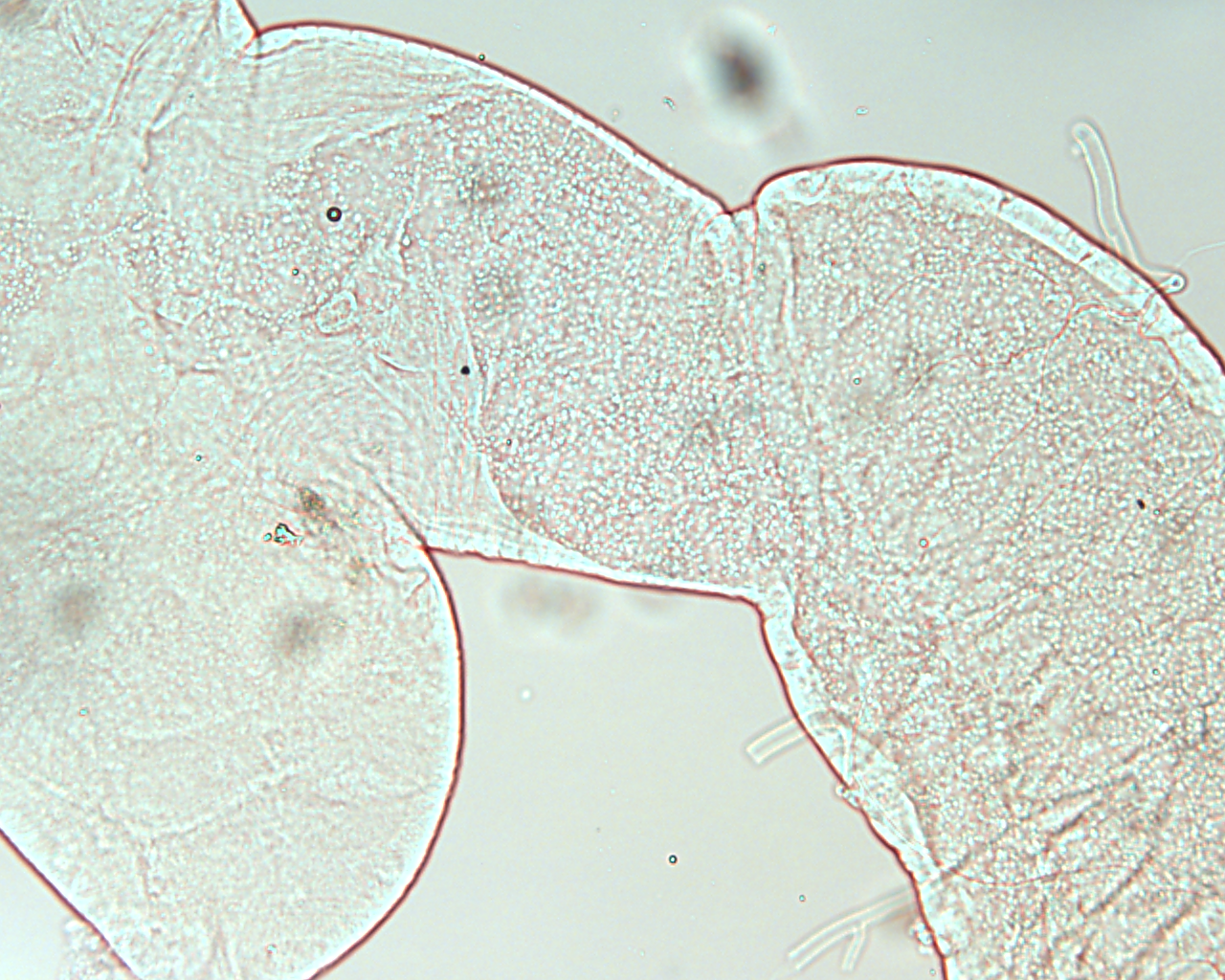

Supplement: Supplementary file 5 — Source data Fig. 1 [file 44319_2025_588_MOESM5_ESM.zip › Figure 1/Figure 1J Image data/Figure 1J.tiff]

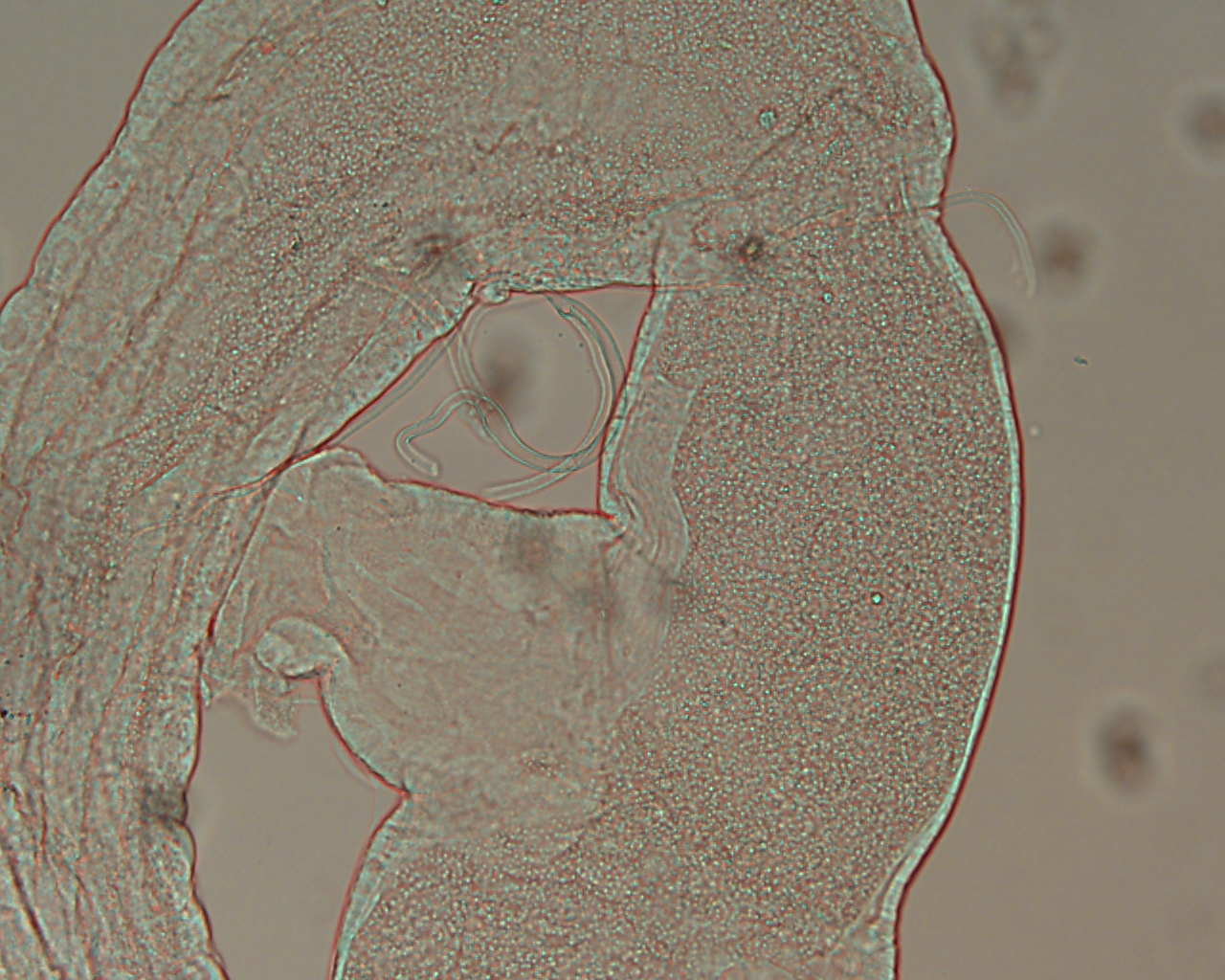

Supplement: Supplementary file 5 — Source data Fig. 1 [file 44319_2025_588_MOESM5_ESM.zip › Figure 1/Figure 1I Image data/Figure 1I.tiff]

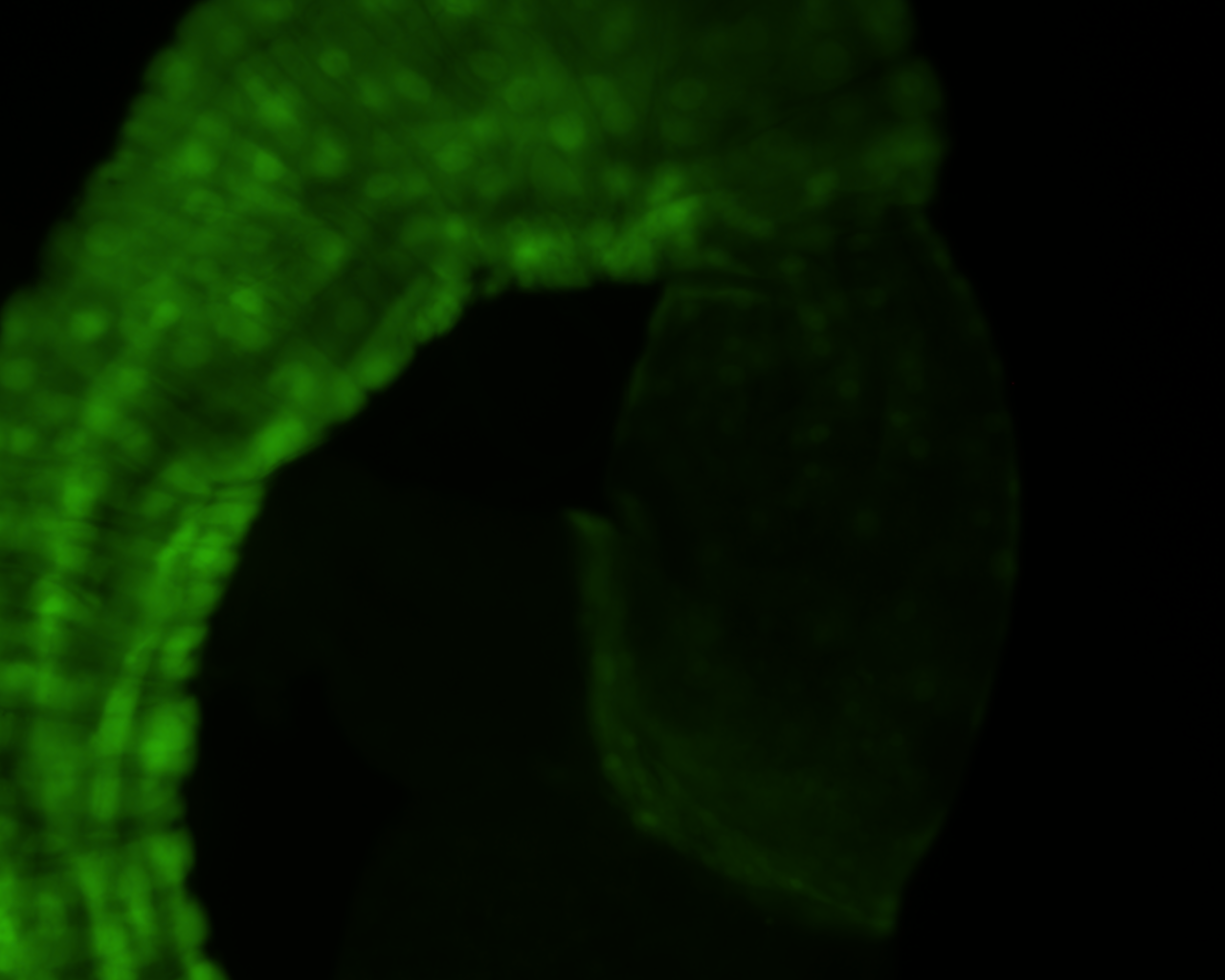

Supplement: Supplementary file 5 — Source data Fig. 1 [file 44319_2025_588_MOESM5_ESM.zip › Figure 1/Figure 1I Image data/Figure 1I''.tiff]

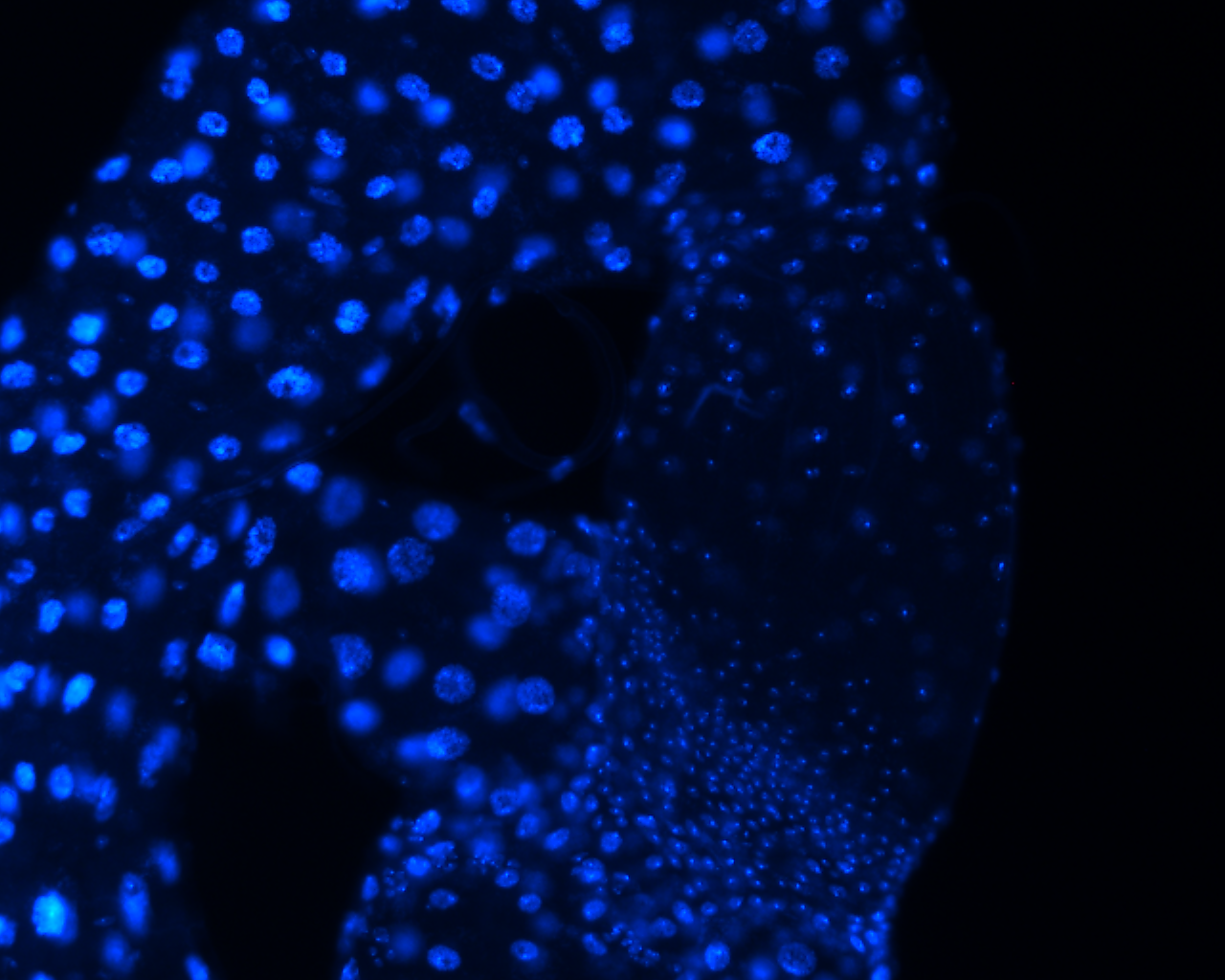

Supplement: Supplementary file 5 — Source data Fig. 1 [file 44319_2025_588_MOESM5_ESM.zip › Figure 1/Figure 1I Image data/Figure 1I'.tiff]

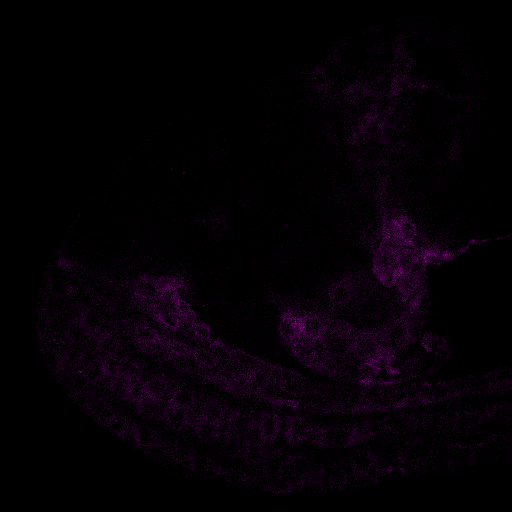

Supplement: Supplementary file 6 — Source data Fig. 2 [file 44319_2025_588_MOESM6_ESM.zip › Figure 2/Figure 2E Image data/2E'.tiff]

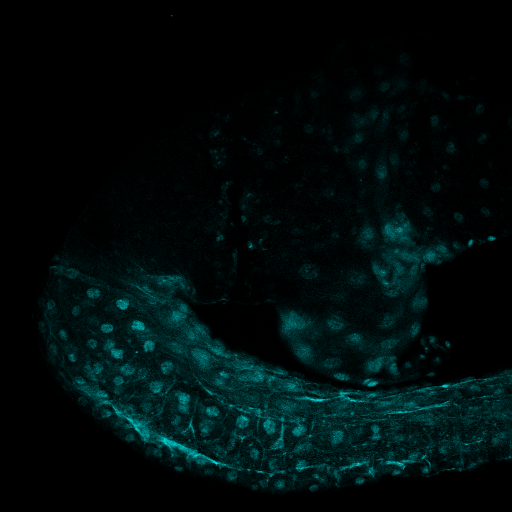

Supplement: Supplementary file 6 — Source data Fig. 2 [file 44319_2025_588_MOESM6_ESM.zip › Figure 2/Figure 2E Image data/2E.tiff]

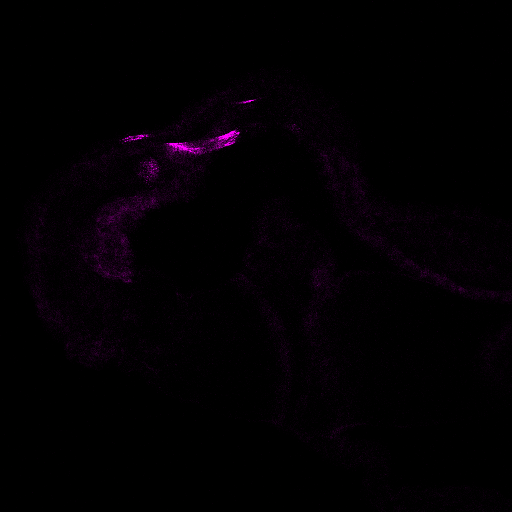

Supplement: Supplementary file 6 — Source data Fig. 2 [file 44319_2025_588_MOESM6_ESM.zip › Figure 2/Figure 2C Image data/2C'.tiff]

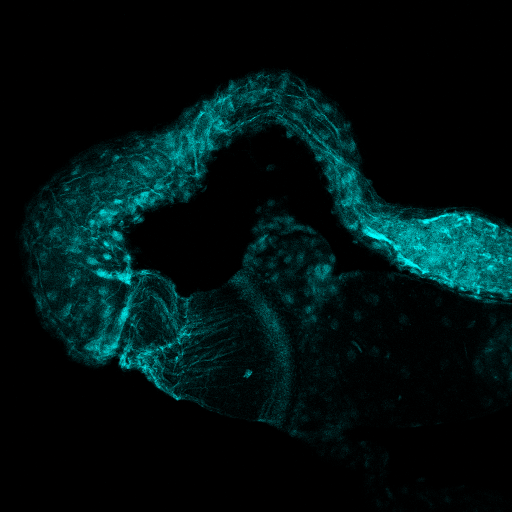

Supplement: Supplementary file 6 — Source data Fig. 2 [file 44319_2025_588_MOESM6_ESM.zip › Figure 2/Figure 2C Image data/2C.tiff]

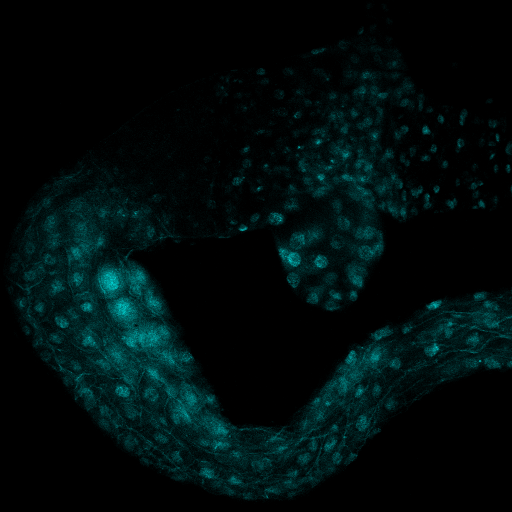

Supplement: Supplementary file 6 — Source data Fig. 2 [file 44319_2025_588_MOESM6_ESM.zip › Figure 2/Figure 2F Image data/2F.tiff]

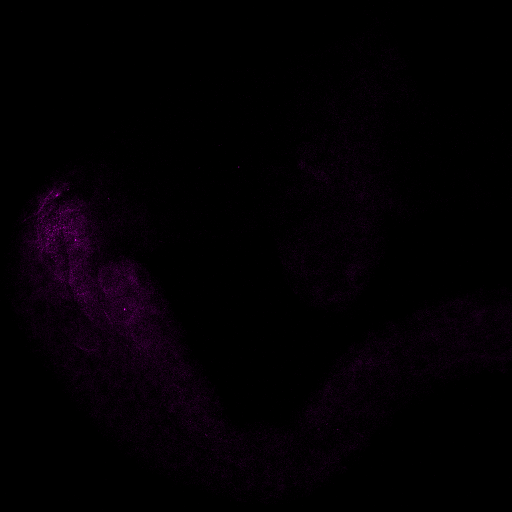

Supplement: Supplementary file 6 — Source data Fig. 2 [file 44319_2025_588_MOESM6_ESM.zip › Figure 2/Figure 2F Image data/2F'.tiff]

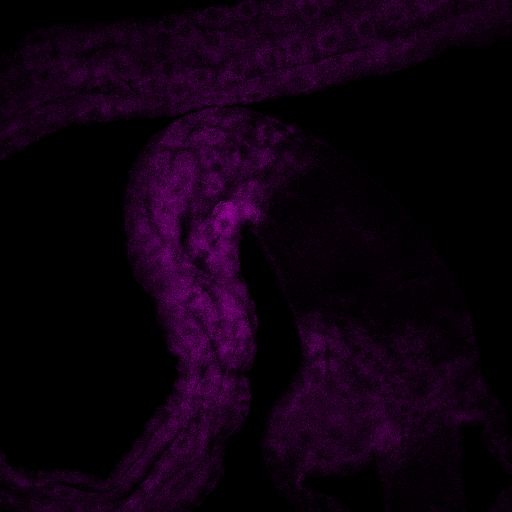

Supplement: Supplementary file 6 — Source data Fig. 2 [file 44319_2025_588_MOESM6_ESM.zip › Figure 2/Figure 2D Image data/2D'.tiff]

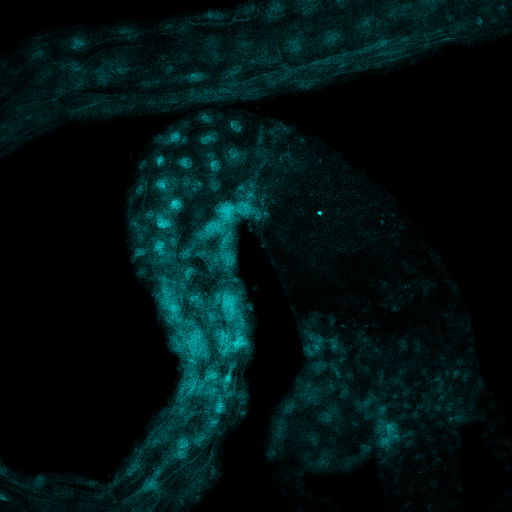

Supplement: Supplementary file 6 — Source data Fig. 2 [file 44319_2025_588_MOESM6_ESM.zip › Figure 2/Figure 2D Image data/2D.tiff]

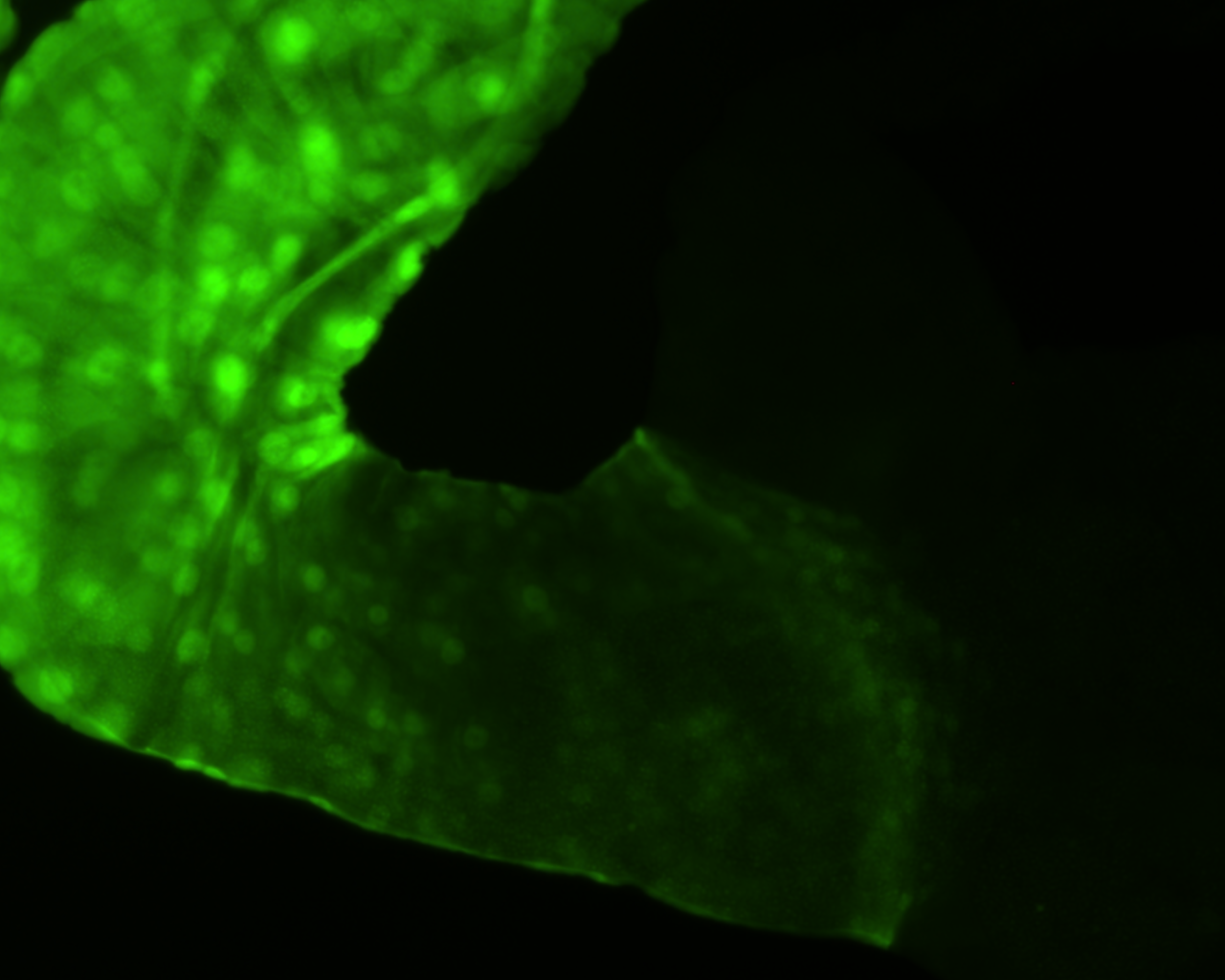

Supplement: Supplementary file 8 — Source data Fig. 4 [file 44319_2025_588_MOESM8_ESM.zip › Figure 4/Figure 4G Image data/Figure 4G''.tiff]

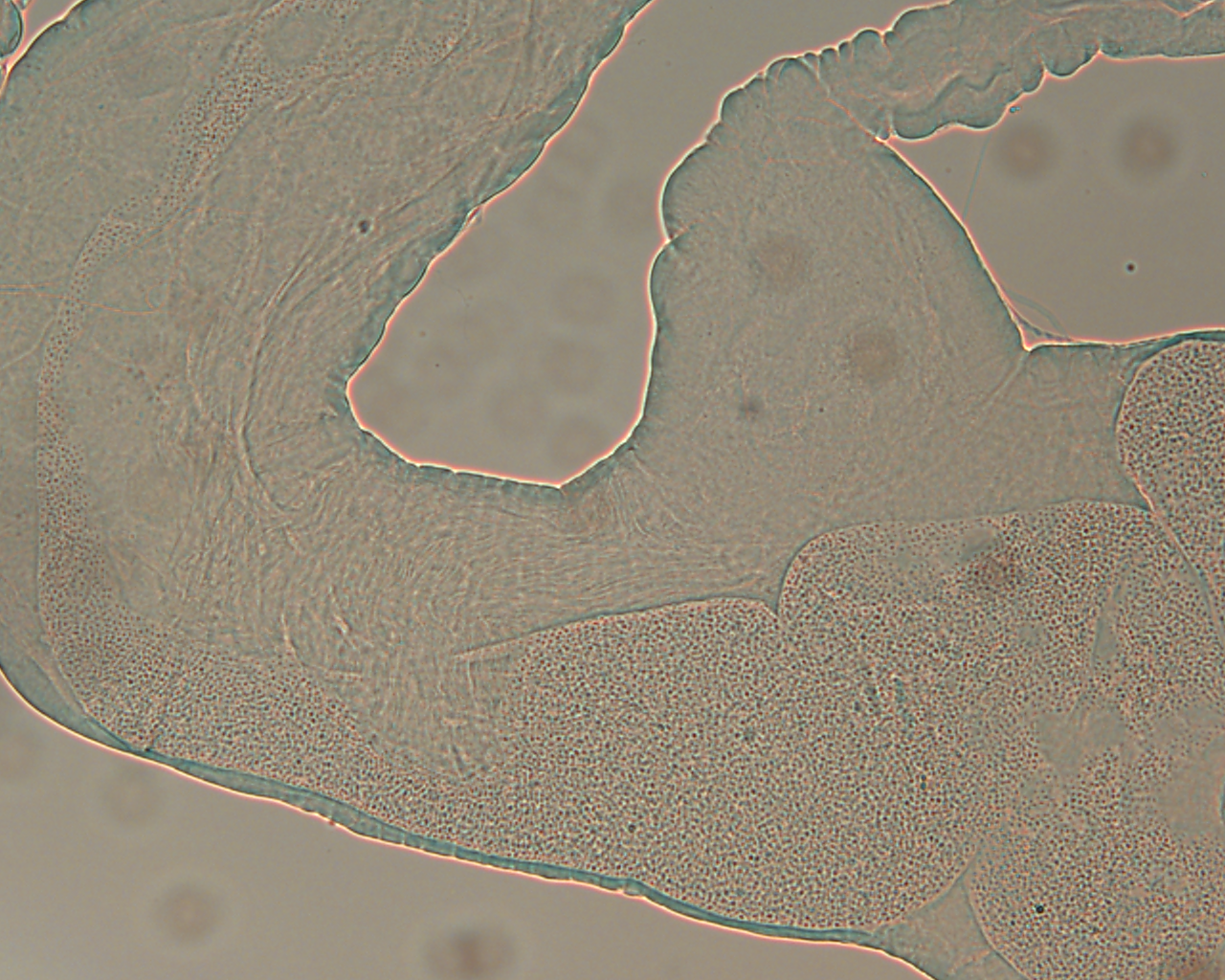

Supplement: Supplementary file 8 — Source data Fig. 4 [file 44319_2025_588_MOESM8_ESM.zip › Figure 4/Figure 4G Image data/Figure 4G.tiff]

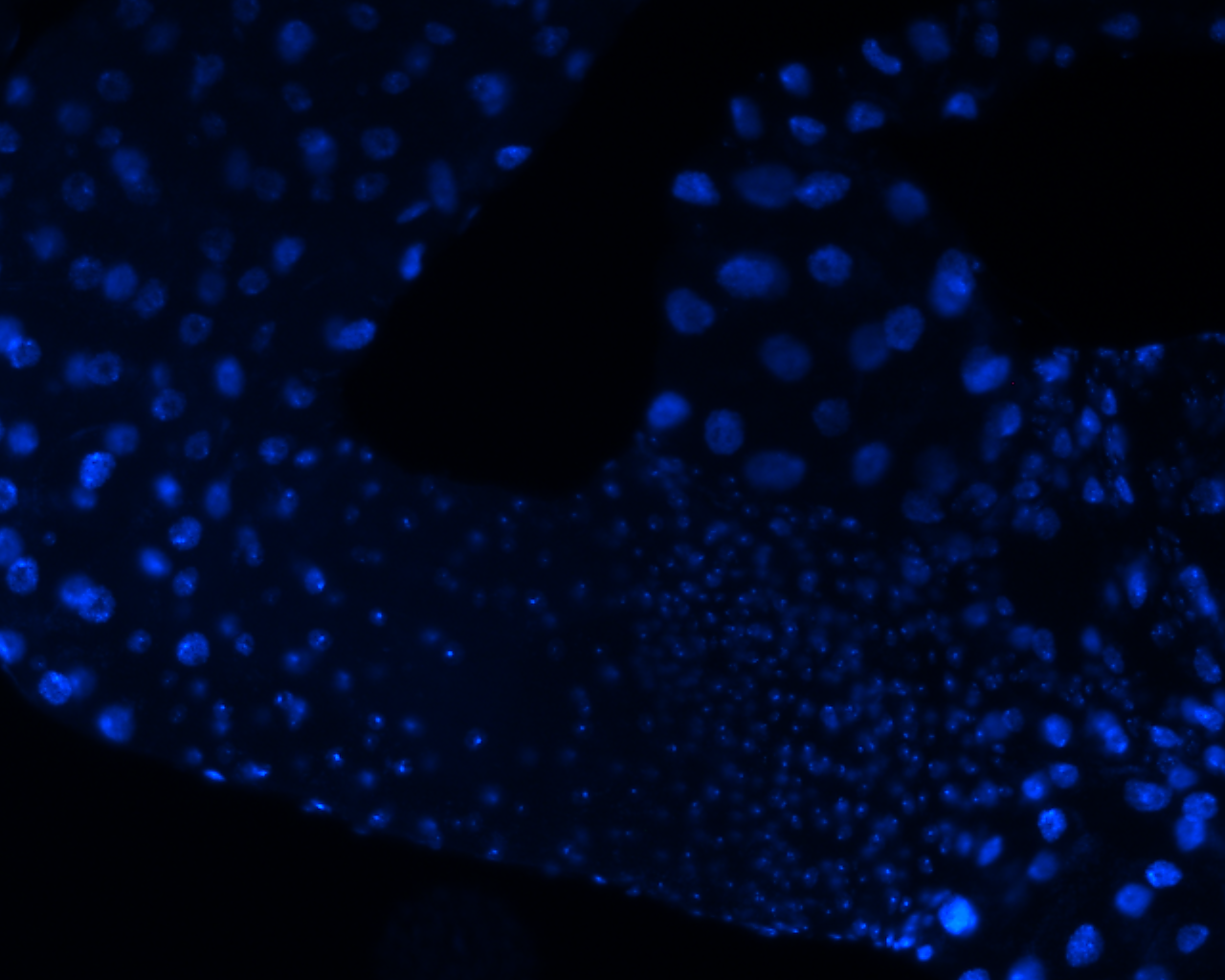

Supplement: Supplementary file 8 — Source data Fig. 4 [file 44319_2025_588_MOESM8_ESM.zip › Figure 4/Figure 4G Image data/Figure 4G'.tiff]

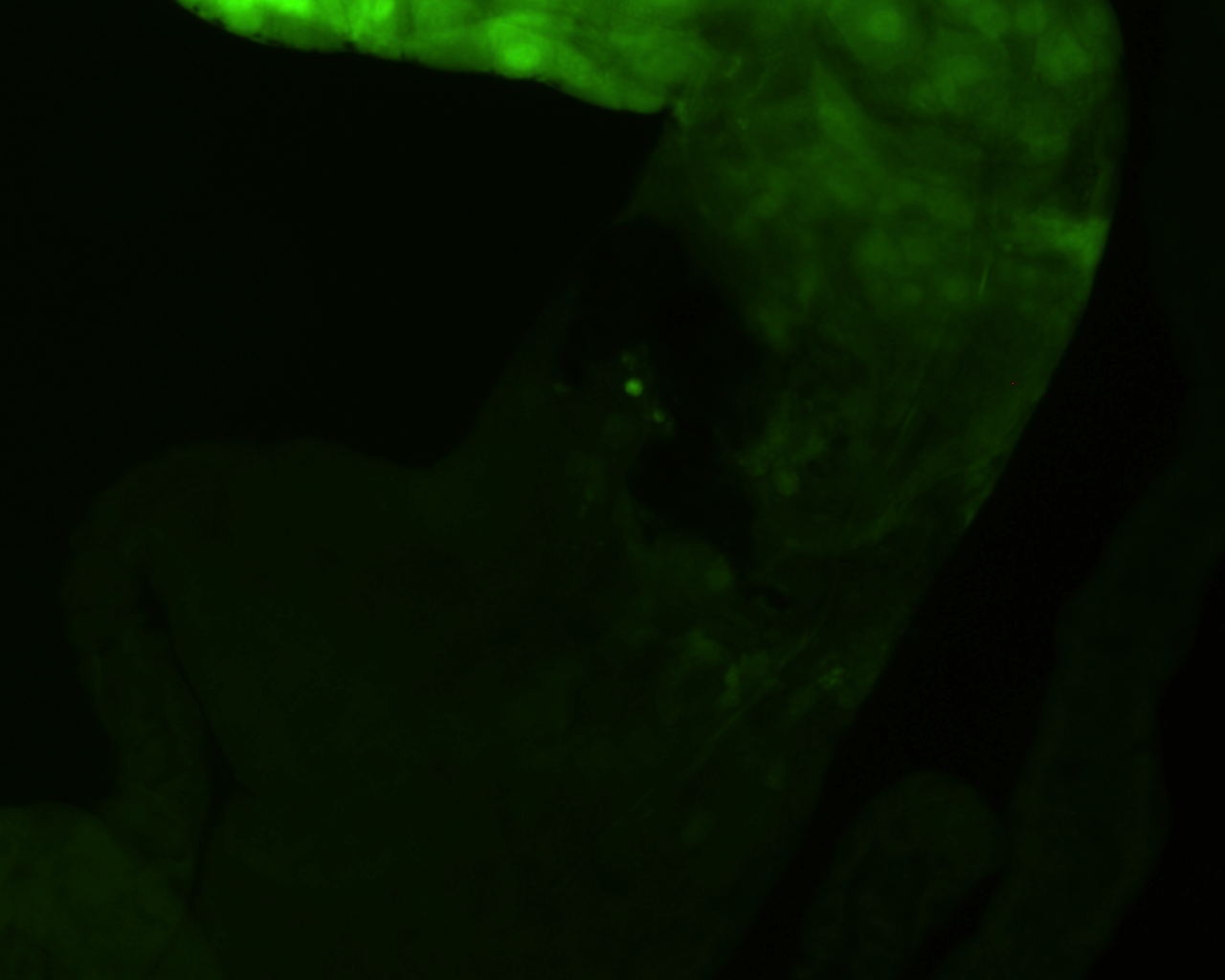

Supplement: Supplementary file 8 — Source data Fig. 4 [file 44319_2025_588_MOESM8_ESM.zip › Figure 4/Figure 4F Image data/Figure 4F''.tiff]

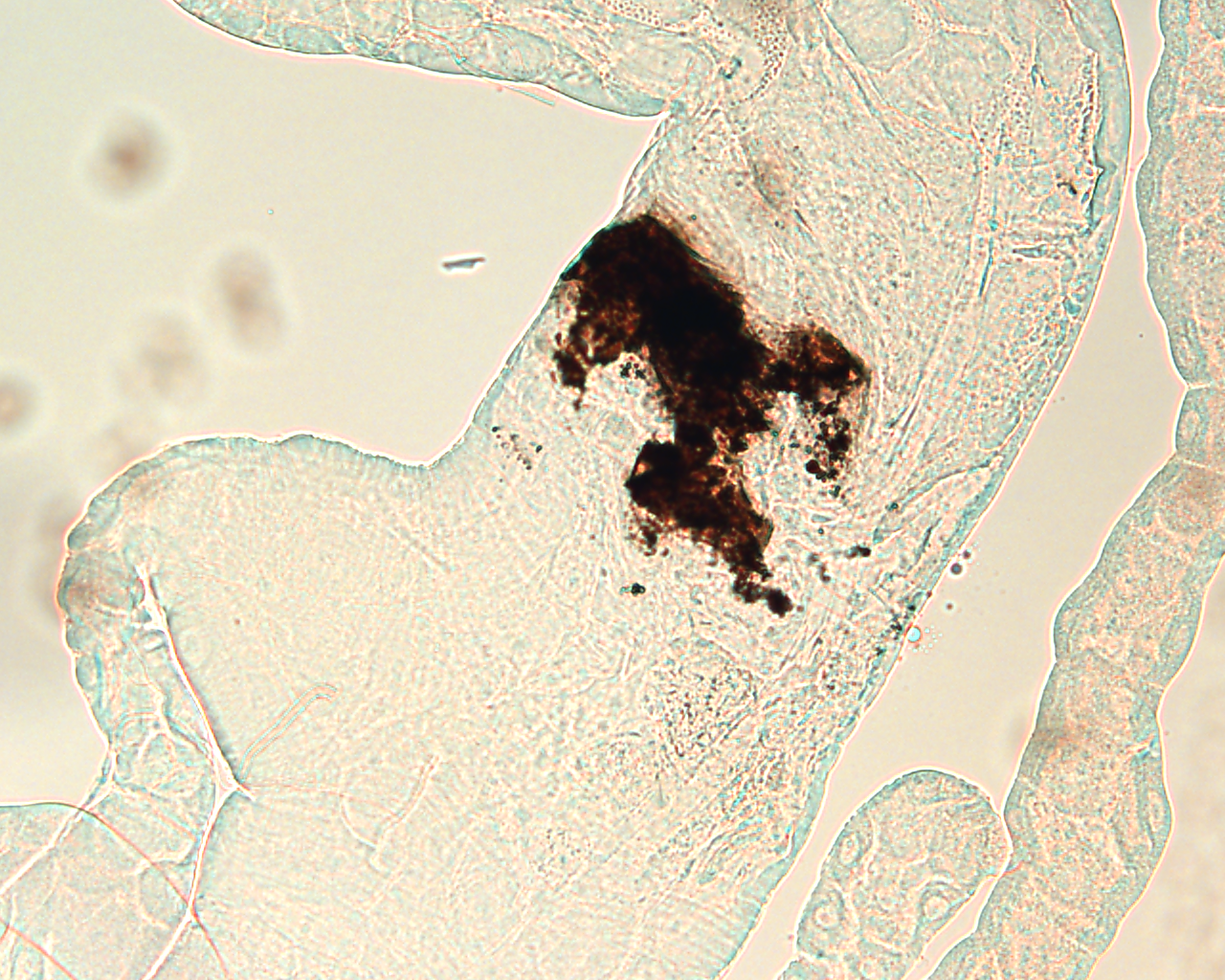

Supplement: Supplementary file 8 — Source data Fig. 4 [file 44319_2025_588_MOESM8_ESM.zip › Figure 4/Figure 4F Image data/Figure 4F.tiff]

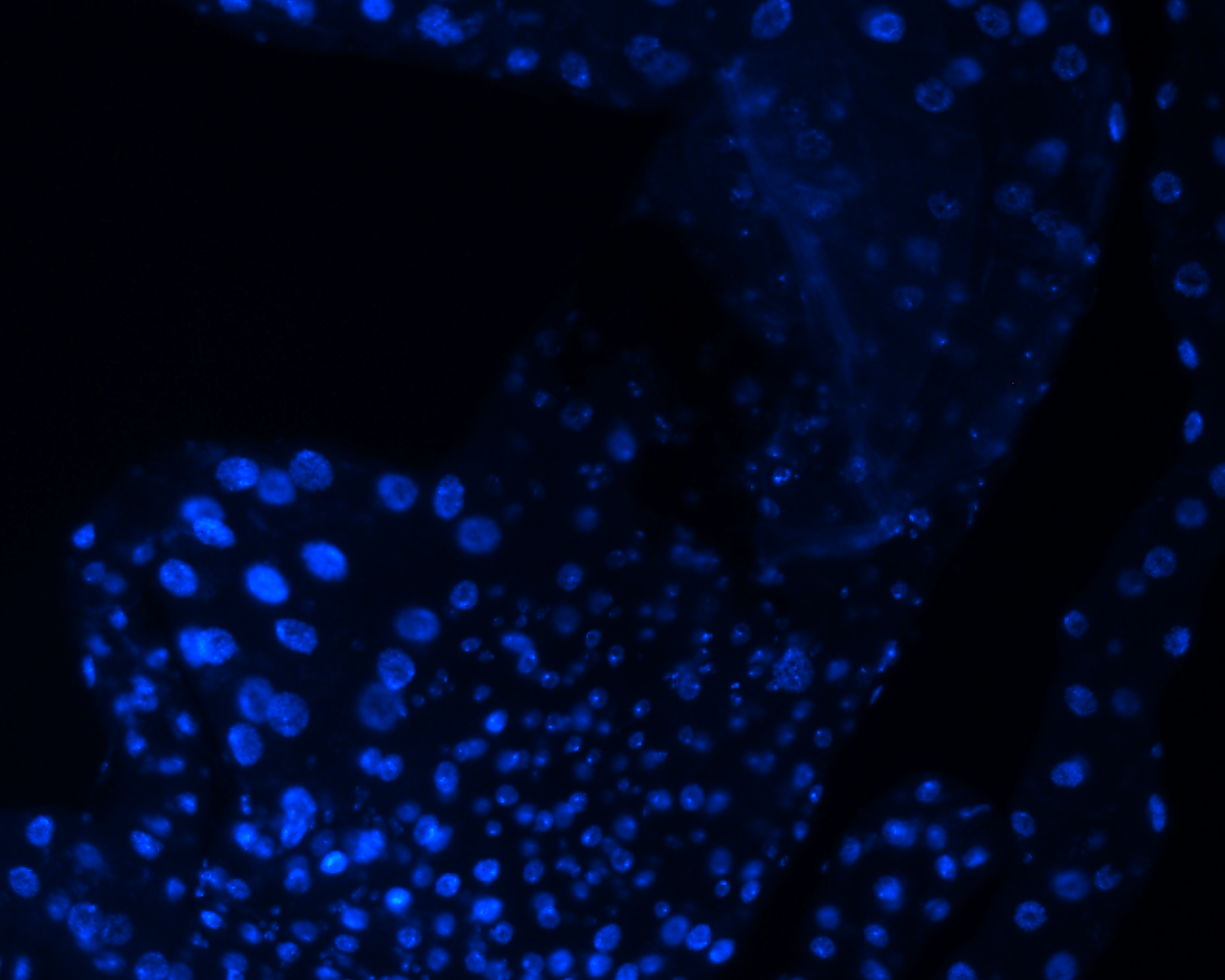

Supplement: Supplementary file 8 — Source data Fig. 4 [file 44319_2025_588_MOESM8_ESM.zip › Figure 4/Figure 4F Image data/Figure 4F'.tiff]

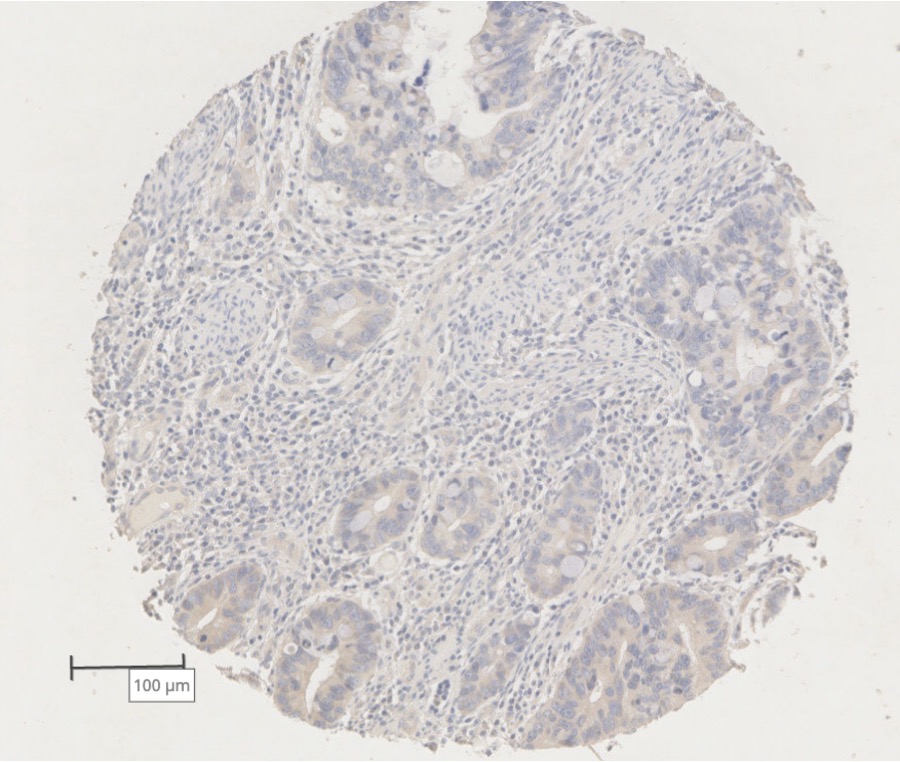

Supplement: Supplementary file 11 — Source data Fig. 7 [file 44319_2025_588_MOESM11_ESM.zip › Figure 7/Figure 7H.jpg]

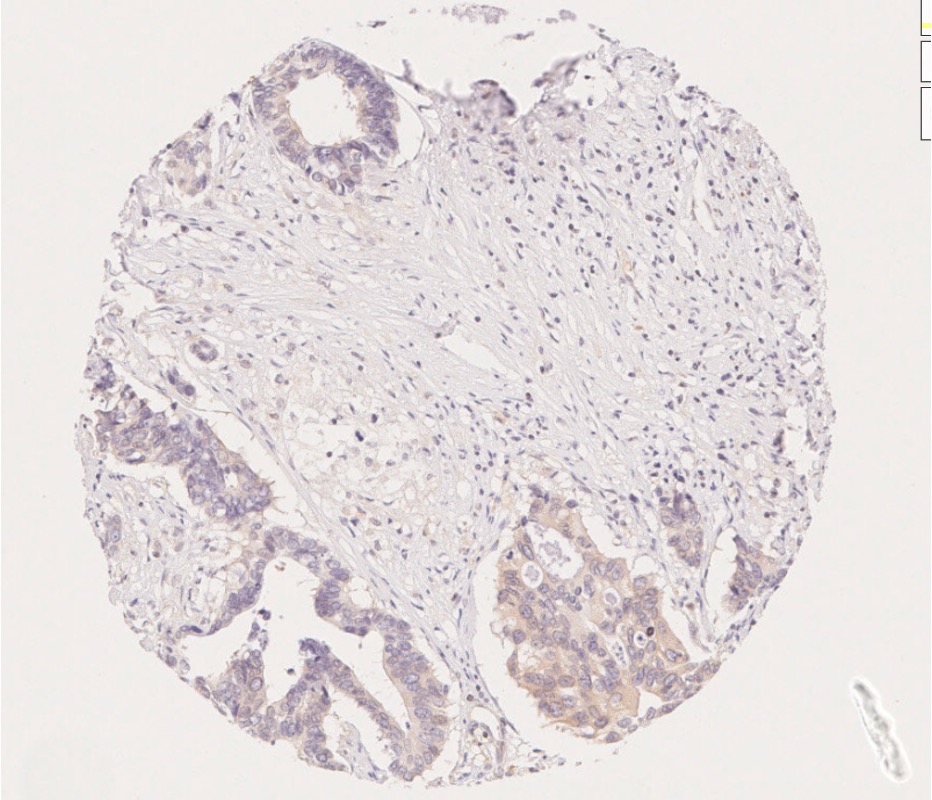

Supplement: Supplementary file 11 — Source data Fig. 7 [file 44319_2025_588_MOESM11_ESM.zip › Figure 7/Figure 7D.jpg]

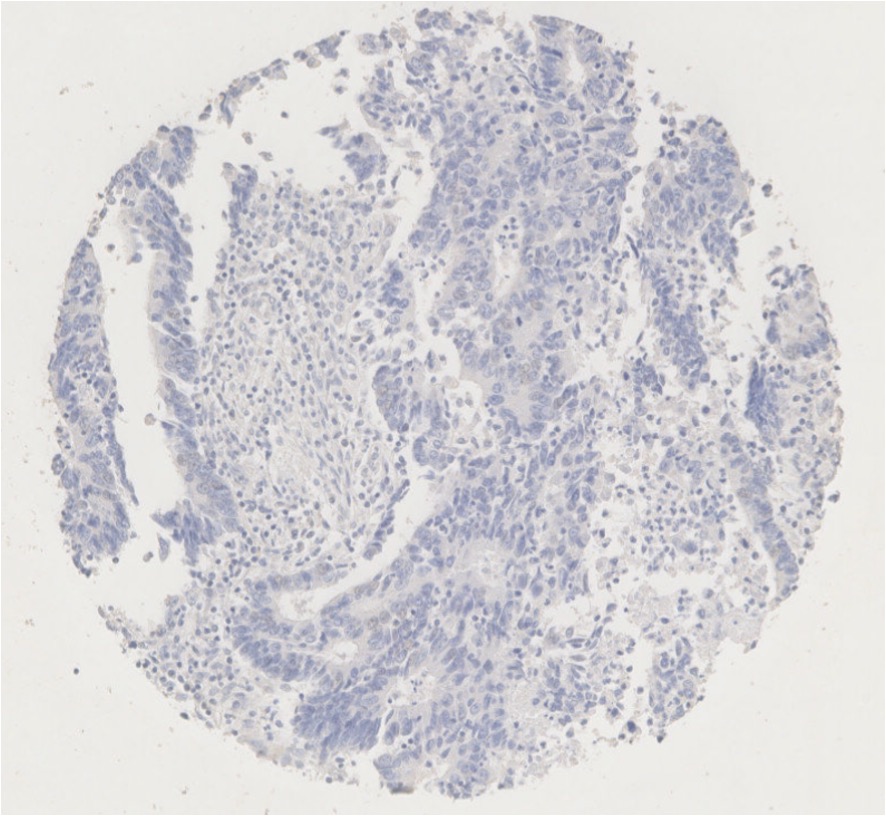

Supplement: Supplementary file 11 — Source data Fig. 7 [file 44319_2025_588_MOESM11_ESM.zip › Figure 7/Figure 7E.jpg]

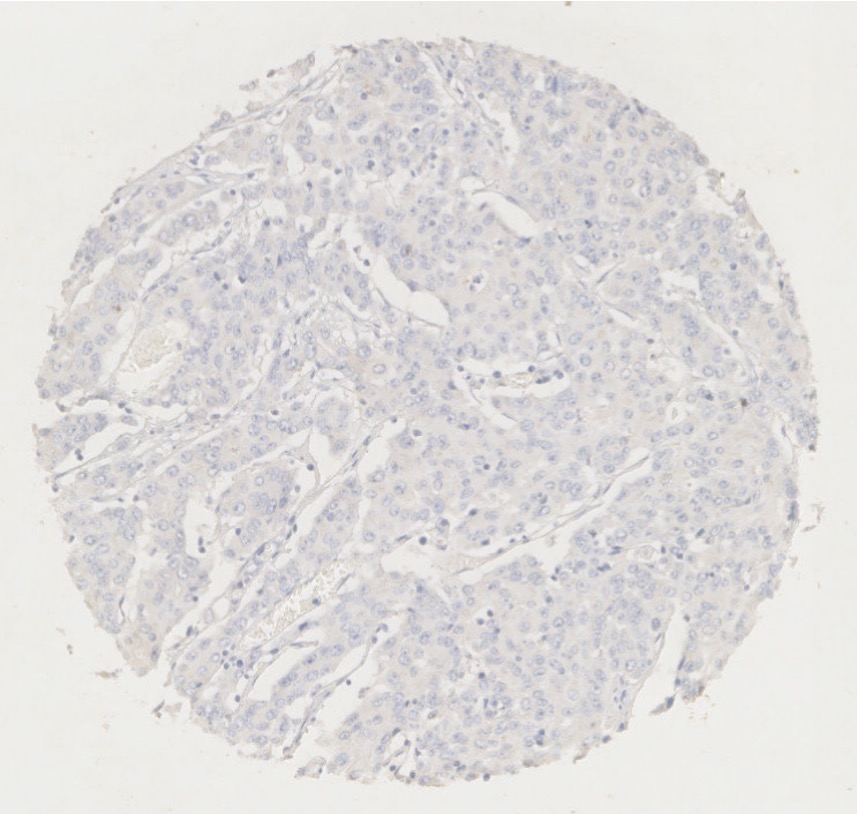

Supplement: Supplementary file 11 — Source data Fig. 7 [file 44319_2025_588_MOESM11_ESM.zip › Figure 7/Figure 7G.jpg]

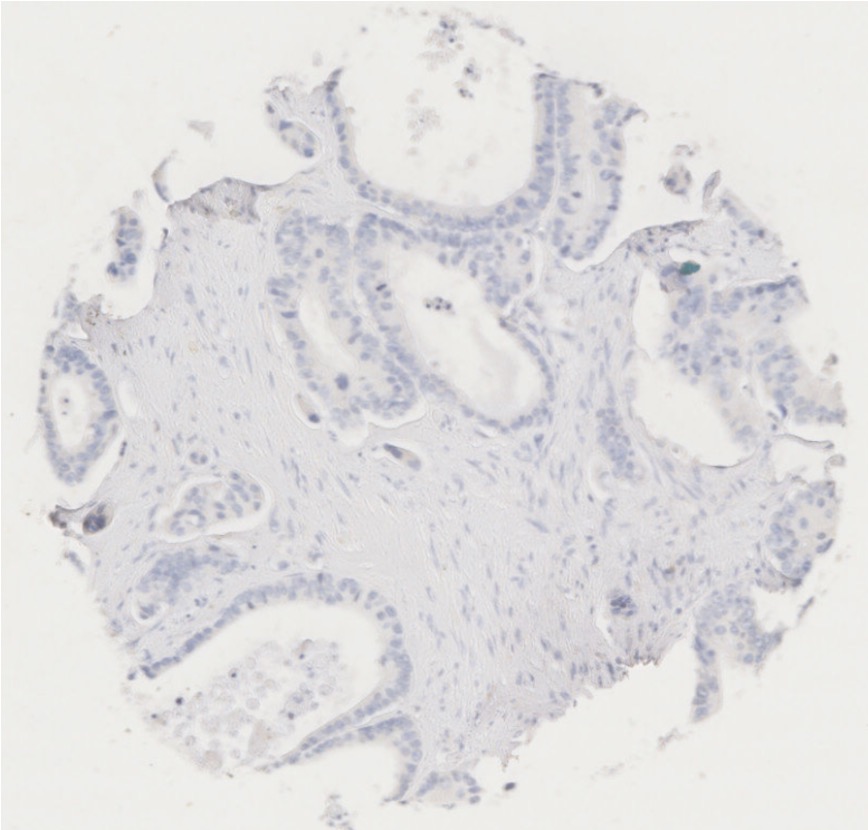

Supplement: Supplementary file 11 — Source data Fig. 7 [file 44319_2025_588_MOESM11_ESM.zip › Figure 7/Figure 7F.jpg]

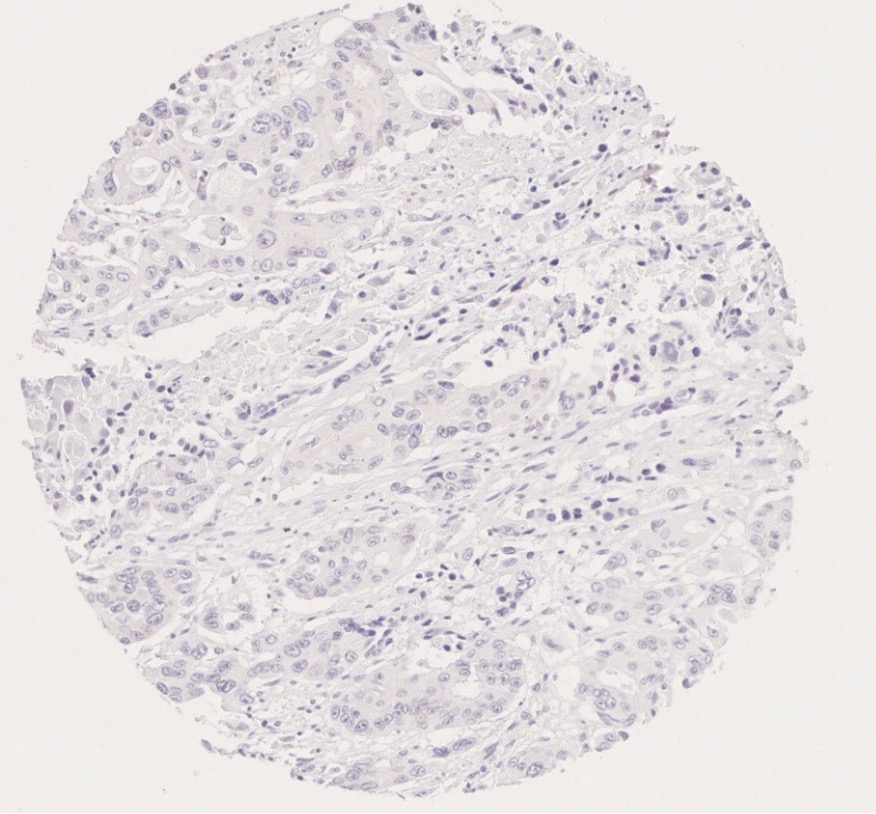

Supplement: Supplementary file 11 — Source data Fig. 7 [file 44319_2025_588_MOESM11_ESM.zip › Figure 7/Figure 7B.jpg]

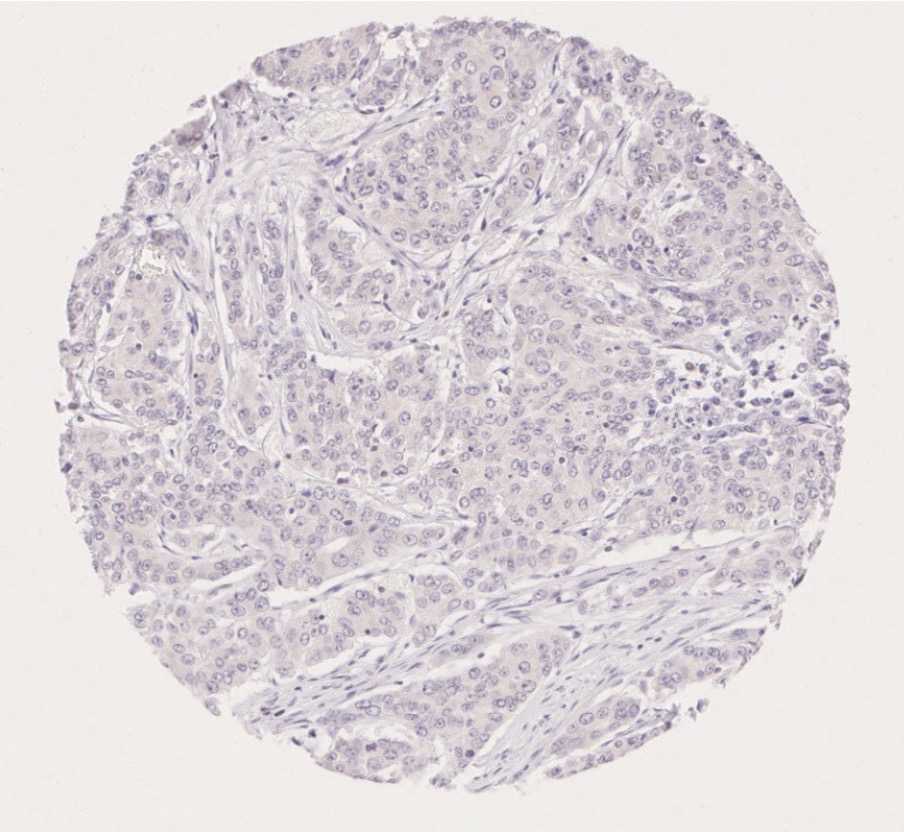

Supplement: Supplementary file 11 — Source data Fig. 7 [file 44319_2025_588_MOESM11_ESM.zip › Figure 7/Figure 7C.jpg]

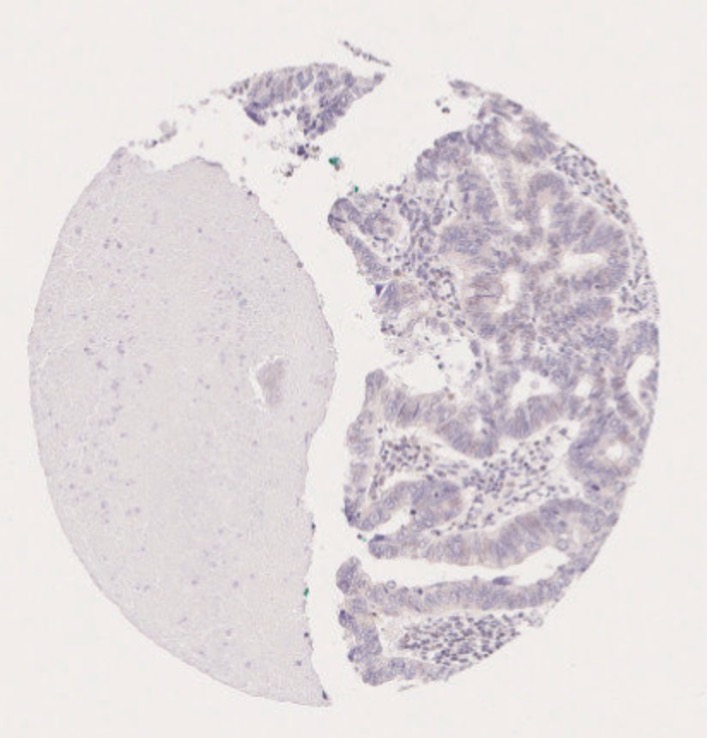

Supplement: Supplementary file 11 — Source data Fig. 7 [file 44319_2025_588_MOESM11_ESM.zip › Figure 7/Figure 7A.jpg]
