# Supplementary material for: WNT signalling promotes NF-κB activation and drug resistance in KRAS-mutant colorectal cancer
Source: EMBO Rep. 2025 Nov 4;26(23):5728–55. doi: 10.1038/s44319-025-00588-1 (PMC12678608; doi:10.1038/s44319-025-00588-1)
Supplement: Supplementary file 13 — Expanded View Figures [file 44319_2025_588_MOESM13_ESM.pdf]

Expanded View Figures

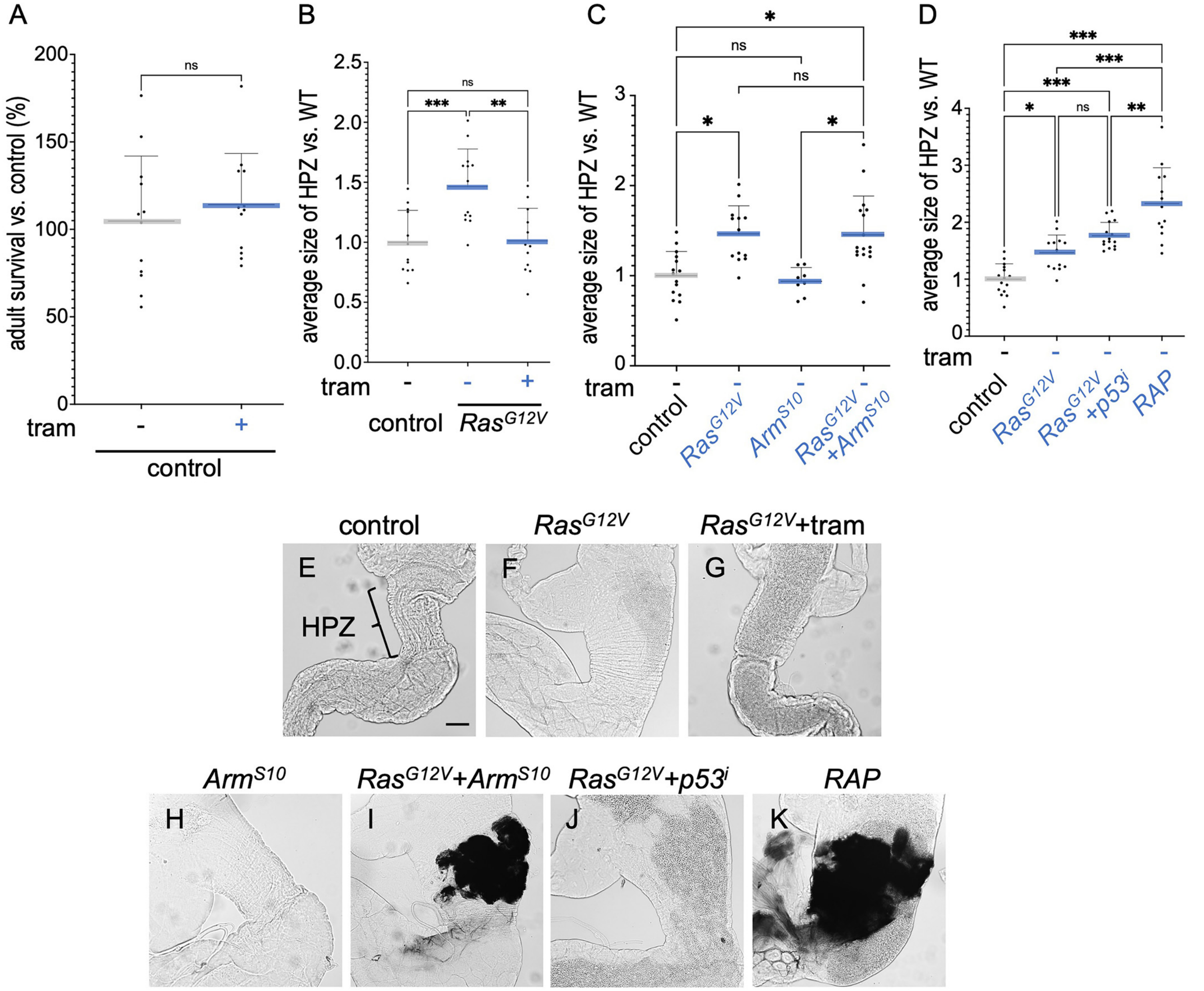

Figure EV1. Overgrowth of HPZ was driven by combined Ras, Wg and p53 alterations.

(**A**) Percent survival of control flies to adulthood relative to control flies was quantified in the presence or absence of trametinib (1 μM). Control (DMSO, $n = 12$; tram, $n = 12$). (**B–D**) The average of the hindgut proliferation zone (HPZ) size was measured by Fiji ImageJ and quantified as relative size to the control hindgut. Control (DMSO, $n = 12$), *Ras*^{G12V} (DMSO, $n = 13$; tram, $n = 12$) (**B**); Control (DMSO, $n = 15$), *Ras*^{G12V} (DMSO, $n = 13$), *Arm*^{S10} (DMSO, $n = 8$), *Ras*^{G12V};*Arm*^{S10} (DMSO, $n = 17$) (**C**); Control (DMSO, $n = 15$), *Ras*^{G12V} (DMSO, $n = 13$), *RAP* (DMSO, $n = 13$), *Ras*^{G12V};*p53*^{i} (DMSO, $n = 15$) (**D**). (**E–K**) Images of the digestive tract of third instar larvae in the presence or absence of trametinib (1 μM). Control (**E**), *Ras*^{G12V} (**F**, **G**), *Arm*^{S10} (**H**), *Ras*^{G12V};*Arm*^{S10} (**I**), *Ras*^{G12V};*p53*^{i} (**J**) and *RAP* (**K**). Scale bar 200 μm. (**A–K**) The experiment was conducted at 27 °C. The statistical tests used to calculate the *P* value are as follows: (**A**) Mann-Whitney test; (**B–D**) one-way ANOVA; NS *P*(>0.12), **P*(0.033), ***P*(0.002) and ****P*(<0.001). All statistical data are summarised in Table EV1. The error bar is a standard deviation (SD), with each point representing biological replicates and numbers (*n*), including three technical replicates.

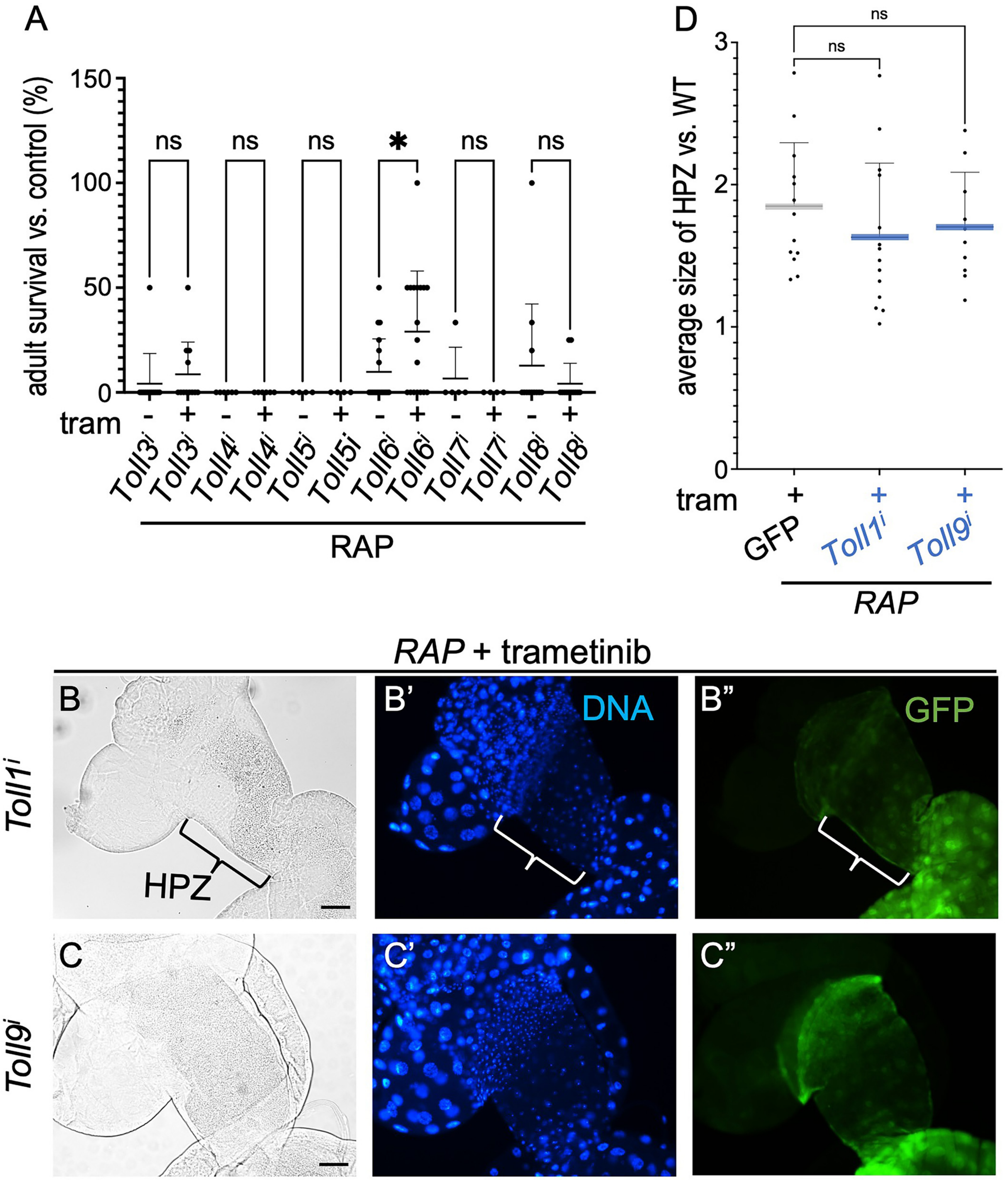
A
adult survival vs. control (%)
150
100
50
0
ns
ns
ns
*
ns
ns
tram
Toll3i
Toll4i
Toll5i
Toll6i
Toll7i
Toll8i
RAP
D
average size of HPZ vs. WT
3
2
1
0
ns
ns
tram
GFP
Toll1i
Toll9i
RAP
RAP + trametinib
B
B'
DNA
B"
GFP
Toll1i
HPZ
C
C'
C"
Toll9i

© The Author(s)

Figure EV2. Administration of NF-κB inhibitors did not suppress drug resistance in *RAP* tumours.

(**A**) Percent survival of transgenic flies to adulthood relative to control flies was quantified in the presence or absence of trametinib (1 μM), *RAP*;*Toll3*^{i} (DMSO, $n = 12$; tram, $n = 12$), *RAP*;*Toll4*^{i} (DMSO, $n = 6$; tram, $n = 6$), *RAP*;*Toll5*^{i} (DMSO, $n = 4$; tram, $n = 4$), *RAP*;*Toll6*^{i} (DMSO, $n = 18$; tram, $n = 18$), *RAP*;*Toll7*^{i} (DMSO, $n = 5$; tram, $n = 4$), *RAP*;*Toll8*^{i} (DMSO, $n = 12$; tram, $n = 12$). (**B**, **C**). Images of the digestive tract of third instar larvae in the presence of trametinib (1 μM), Scale bar 200 μm. (**D**) Average hindgut proliferation zone (HPZ) size was measured by Fiji ImageJ and quantified as relative size to the control hindgut. *RAP*;*GFP* (tram, $n = 13$), *RAP*;*Toll1*^{i} (tram, $n = 14$), *RAP*;*Toll9*^{i} (tram, $n = 10$). (**A**–**D**) The experiment was conducted at 29 °C. The statistical tests used to calculate the *P* value are as follows: (**A**, **B**) one-way ANOVA; NS *P*(>0.12), **P*(0.033), ***P*(0.002) and ****P*(<0.001). All statistical data are summarised in Table EV1. The error bar is a standard deviation (SD), with each point representing biological replicates and numbers (*n*), including three technical replicates.

© The Author(s)

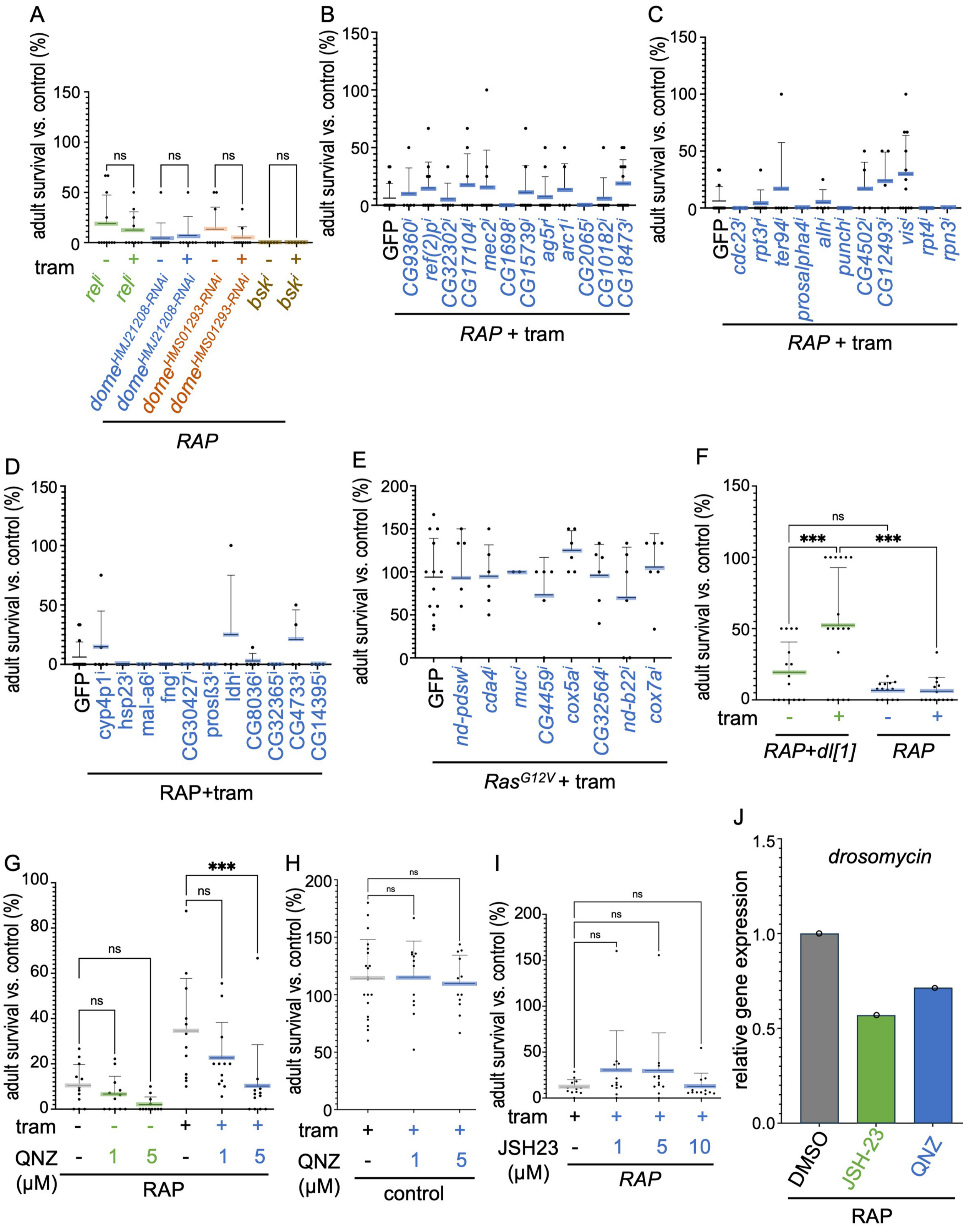
A
adult survival vs. control (%)
ns
tram
rel^i
dome^HMJ21208-RNAi
dome^HMS01293-RNAi
bsk^i
RAP
B
GFP
CG9360^i
ref(2)p^i
CG32302^i
CG17104^i
mec2^i
CG1698^i
CG15739^i
ag5r^i
arc1^i
CG2065^i
CG10182^i
CG18473^i
RAP + tram
C
GFP
cdc23^i
rpt3r^i
ter94^i
prosalpha4^i
alh^i
punch^i
CG4502^i
CG12493^i
vis^i
rpt4^i
rpn3^i
RAP + tram
D
GFP
cyp4p1^i
hsp23^i
mal-a6^i
fng^i
CG30427^i
prosß3^i
ldh^i
CG8036^i
CG32365^i
CG4733^i
CG14395^i
RAP+tram
E
GFP
nd-pdsw^i
cda4^i
muc^i
CG4459^i
cox5a^i
CG32564^i
nd-b22^i
cox7a^i
Ras^G12V + tram
F
***
RAP+dl[1]
RAP
G
QNZ (μM)
RAP
H
control
I
JSH23 (μM)
RAP
J
drosomycin
relative gene expression
DMSO
JSH-23
QNZ
RAP

Figure EV3. Screening for factors that influence drug response.

(**A–E**) Percent survival of transgenic flies to adulthood relative to control flies was quantified in the presence or absence of trametinib (1 µM). All flies driven by *byn-GAL4*: (**A**) *RAP;rel^i^* (DMSO, $n = 11$; tram, $n = 10$), *RAP;dome^i^* (HMJ21208) (DMSO, $n = 11$; tram, $n = 7$), *RAP;dome^i^* (HMS01293) (DMSO, $n = 10$; tram, $n = 10$), *RAP;bsk^i^* (DMSO, $n = 6$; tram, $n = 6$); (**B**) *RAP;GFP* (tram, $n = 14$), *RAP;CG9360^i^* (tram, $n = 5$), *RAP;ref(2)p^i^* (tram, $n = 10$), *RAP;CG32302^i^* (tram, $n = 6$), *RAP;CG17104^i^* (tram, $n = 8$), *RAP;mec2^i^* (tram, $n = 10$), *RAP;CG1698^i^* (tram, $n = 5$), *RAP;CG15739^i^* (tram, $n = 9$), *RAP;ag5r^i^* (tram, $n = 11$), *RAP;arc^i^* (tram, $n = 6$), *RAP;CG2065^i^* (tram, $n = 6$), *RAP;CG10182^i^* (tram, $n = 8$), *RAP;18473^i^* (tram, $n = 12$); (**C**) *RAP;GFP* (tram, $n = 14$), *RAP;cdc23^i^* (tram, $n = 3$), *RAP;rpt3r^i^* (tram, $n = 8$), *RAP;ter94^i^* (tram, $n = 6$), *RAP;prosalpha4^i^* (tram, $n = 4$), *RAP;alh^i^* (tram, $n = 5$), *RAP;punch^i^* (tram, $n = 6$), *RAP;CG4502^i^* (tram, $n = 5$), *RAP;CG12493^i^* (tram, $n = 5$), *RAP;vis^i^* (tram, $n = 12$), *RAP;rpt4^i^* (tram, $n = 5$), *RAP;rpn3^i^* (tram, $n = 2$); (**D**) *RAP;GFP* (tram, $n = 14$), *RAP;cyp4p1^i^* (tram, $n = 6$), *RAP;hsp23^i^* (tram, $n = 6$), *RAP;mal-a6^i^* (tram, $n = 4$), *RAP;fng^i^* (tram, $n = 6$), *RAP;CG30427^i^* (tram, $n = 3$), *RAP;prosbeta3^i^* (tram, $n = 4$), *RAP;ldh^i^* (tram, $n = 4$), *RAP;CG8036^i^* (tram, $n = 5$), *RAP;CG32365^i^* (tram, $n = 4$), *RAP;CG4733^i^* (tram, $n = 4$), *RAP;CG14395^i^* (tram, $n = 4$); (**E**) Ras^{G12V}*;GFP* (tram, $n = 13$), Ras^{G12V}*;nd-pdsw^i^* (tram, $n = 6$), Ras^{G12V}*;cda4^i^* (tram, $n = 6$), Ras^{G12V}*;muc^i^* (tram, $n = 2$), Ras^{G12V}*;CG4459^i^* (tram, $n = 5$), Ras^{G12V}*;cox5a^i^* (tram, $n = 6$), Ras^{G12V}*;CG32564^i^* (tram, $n = 6$), Ras^{G12V}*;nd-b22^i^* (tram, $n = 6$), Ras^{G12V}*;cox7a^i^* (tram, $n = 6$). (**F–I**) Percent survival of transgenic flies to adulthood relative to control flies was quantified in the presence or absence of trametinib (1 µM), QNZ (EVP4593) or JSH-23. (**F**) *RAP;dl[1]* (DMSO, $n = 16$; tram, $n = 18$) and *RAP* (DMSO, $n = 14$; tram, $n = 14$); (**G–I**) *RAP* (DMSO, $n = 12$; tram, $n = 12$; 1 µM QNZ, $n = 12$; 5 µM QNZ, $n = 12$; tram + 1 µM QNZ, $n = 12$; tram + 5 µM QNZ, $n = 12$) (**G**); control (tram, $n = 20$; tram + 1 µM QNZ, $n = 12$; tram + 5 µM QNZ, $n = 12$) (**H**); *RAP* (tram, $n = 12$; tram + 1 µM JSH-23, $n = 12$; tram + 5 µM JSH-23, $n = 12$; tram + 10 µM JSH-23, $n = 12$) (**I**). (**J**) Expression levels of *drosomycin* were quantified for each condition by quantitative RT-PCR, QNZ (5 µM), JSH-23 (5 µM). (**A–D**, **F**) The experiments were conducted at 29 °C. (**E**, **G–I**) The experiments were conducted at 29 °C. The statistical tests used to calculate the *P* value are as follows: (**A**, **F–I**) one-way ANOVA; NS *P*(>0.12), **P*(0.033) and ****P*(<0.001). All statistical data are summarised in Table EV1. The error bar is a standard deviation (SD), with each point representing biological replicates and numbers (*n*), including three technical replicates.

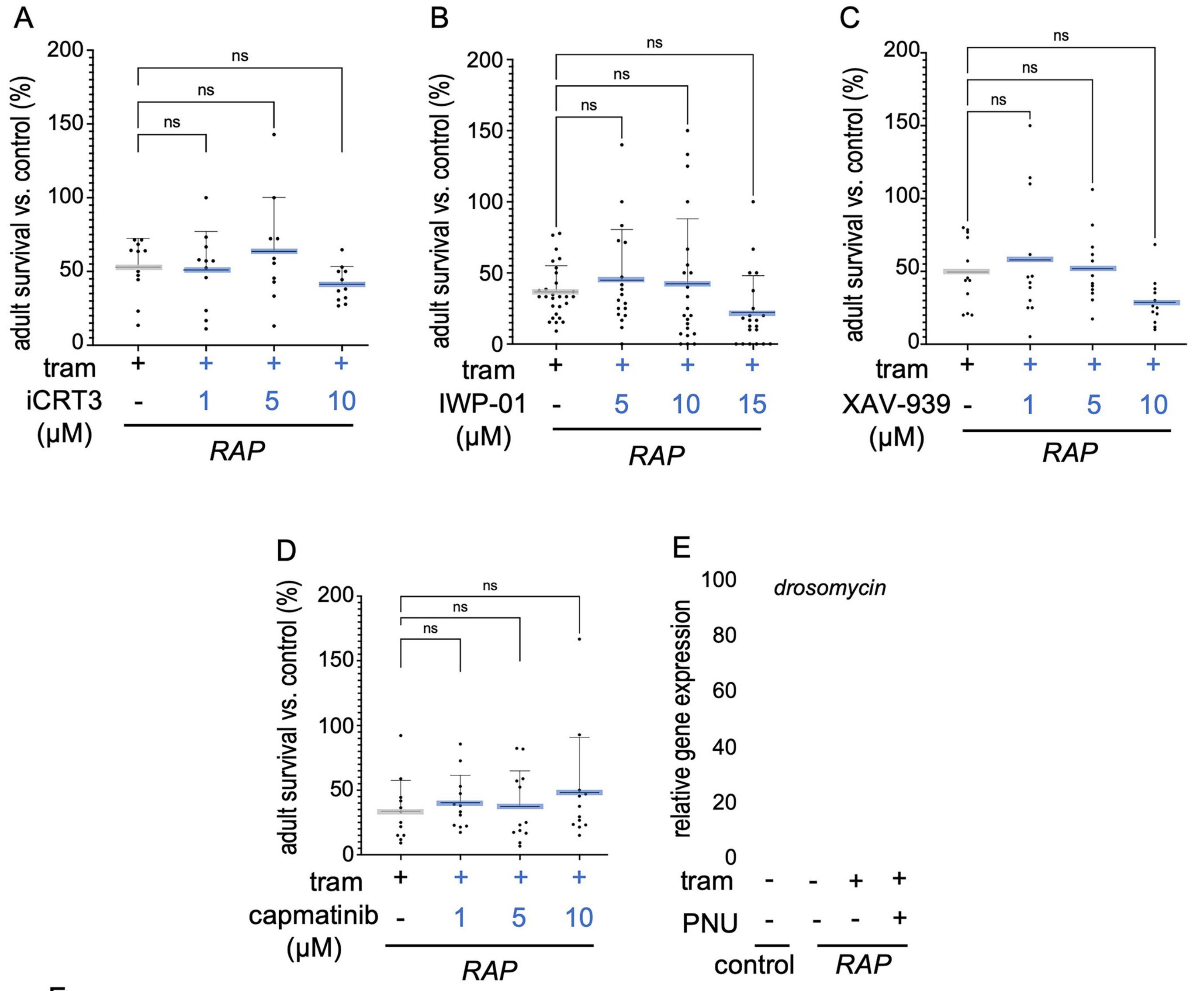

F

Line	Gain of function genes	Loss of function genes
CPCT006	*Ras*^{G12V}	*apc; p53; ago; shg; put; p38a; ft; brm*
CPCT018	*Ras*^{G12V}	*apc; p53; pten; smox; tefu; rhoGAPp190; nejire; upf1*
CPCT029	*Ras*^{G12V}*; chico*	*apc; p53; Med; smox; trr; fur2; CG4238; PI4KIIIalpha, mus81*
CPCT045	*Ras*^{G12V}*; pvr*	*apc; p53; smox; nos; CG13344; pc; rad51C; DNApol-eta; lrp1*
CPCT050	*Ras*^{G12V}	*apc; p53; debcl; scat; wdb; ird1*
RAPp1	*Ras*^{G12V}	*apc; p53; ago; wts; CG7742; Atg2*
RAPp2	*Ras*^{G12V}	*apc; p53; vrp1; ry; khc-73*

 © The Author(s)

Figure EV4. A screening for trametinib and WNT inhibitors drug combination in *RAP* hindgut tumours.

(**A–D**) Percent survival of transgenic *RAP* flies to adulthood relative to control flies was quantified in the presence or absence of trametinib (1 μM), iCRT3, IWP-01, XAV-939 or Capmatinib. *RAP* (tram, $n = 11$; tram + 1 μM iCRT3, $n = 11$; tram + 5 μM iCRT3, $n = 10$; tram + 10 μM iCRT3, $n = 11$) (**A**); *RAP* (tram, $n = 28$; tram + 5 μM IWP-01, $n = 18$; tram + 10 μM IWP-01, $n = 22$; tram + 15 μM IWP-01, $n = 21$) (**B**); *RAP* (tram, $n = 12$; tram + 1 μM XAV-939, $n = 12$; tram + 5 μM XAV-939, $n = 12$; tram + 10 μM XAV-939, $n = 12$) (**C**); *RAP* (tram, $n = 12$; tram + 1 μM Capmatinib, $n = 12$; tram + 5 μM Capmatinib, $n = 12$; tram + 10 μM Capmatinib, $n = 12$) (**D**). (**E**) The expression levels of *drosomycin* among each genotype in the presence or absence of trametinib (1 μM) or PNU-74654 (1 μM) were detected by quantitative RT-PCR, $n = 3$. (**E**) *control* and *RAP*. (**A–E**) The experiment was conducted at 27 °C. The statistical tests used to calculate the *P* value are as follows: (**A–D**) one-way ANOVA; NS *P*(>0.12), **P*(0.033), ***P*(0.002) and ****P*(<0.001). All statistical data are summarised in Table EV1. The error bar is a standard deviation (SD), with each point representing biological replicates and numbers (*n*), including three technical replicates. (**F**) A summary of mutations in patient-specific CRC models. These patient-specific fly avatars come from a fly-to-bedside study (CPCTs). and TCGA (*RAPp1*, *RAPp2*).

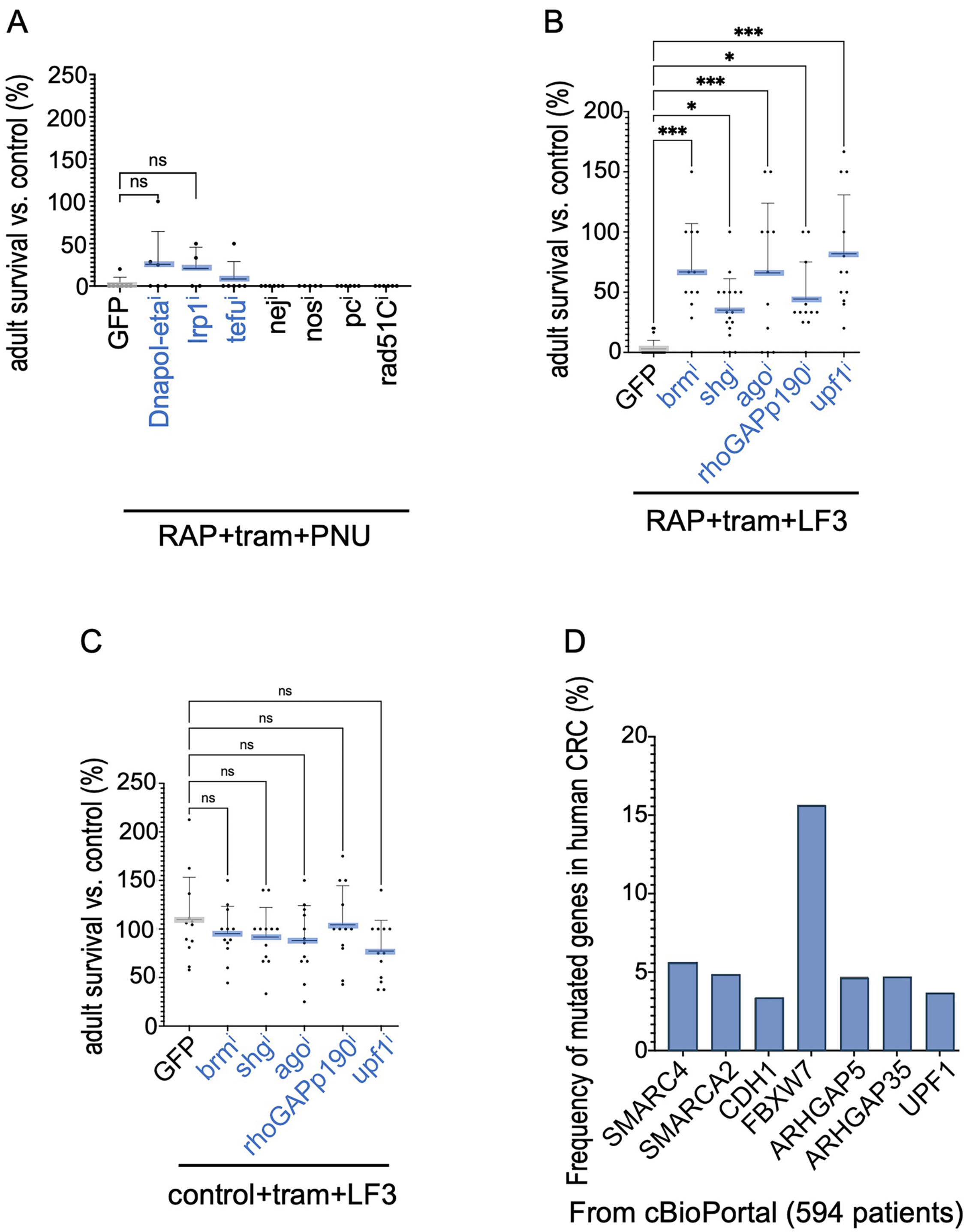

Figure EV5. Regulators for the combination of trametinib and LF3 in CRC tumours.

(**A–C**) Percent survival of transgenic flies to adulthood relative to control flies was quantified in the presence or absence of trametinib (1 μM), PNU-74654 (1 μM) or LF3 (10 μM). *RAP;GFP* (tram + PNU, *n* = 7), *RAP;DNApol-eta^i* (tram+PNU, *n* = 6); *RAP;lrp1^i* (tram + PNU, *n* = 4) (**A**). *RAP;GFP* (tram + LF3, *n* = 19), *RAP;brm^i* (tram + LF3, *n* = 12), *RAP;ago^i* (tram + LF3, *n* = 11), *RAP;shg^i* (tram + LF3, *n* = 17), *RAP;upf1^i* (tram + LF3, *n* = 12), *RAP;rhoGAPp190^i* (tram + LF3, *n* = 12) (**B**); *control* (tram + LF3, *n* = 12), *brm^i* (tram + LF3, *n* = 12), *ago^i* (tram + LF3, *n* = 12), *shg^i* (tram + LF3, *n* = 12), *upf1^i* (tram + LF3, *n* = 12), *rhoGAPp190^i* (tram + LF3, *n* = 12) (**C**). (**D**) The graph showed the frequency of mutated genes in human CRC. These data from cBioPortal include 594 patients. (**A–C**) The experiment was conducted at 29 °C. The statistical tests used to calculate the *P* value are as follows: (**A–C**) one-way ANOVA; NS *P*(>0.12), **P*(0.033), ***P*(0.002) and ****P*(<0.001). All statistical data are summarised in Table EV1. The error bar is a standard deviation (SD), with each point representing biological replicates and numbers (*n*), including three technical replicates.

 © The Author(s)

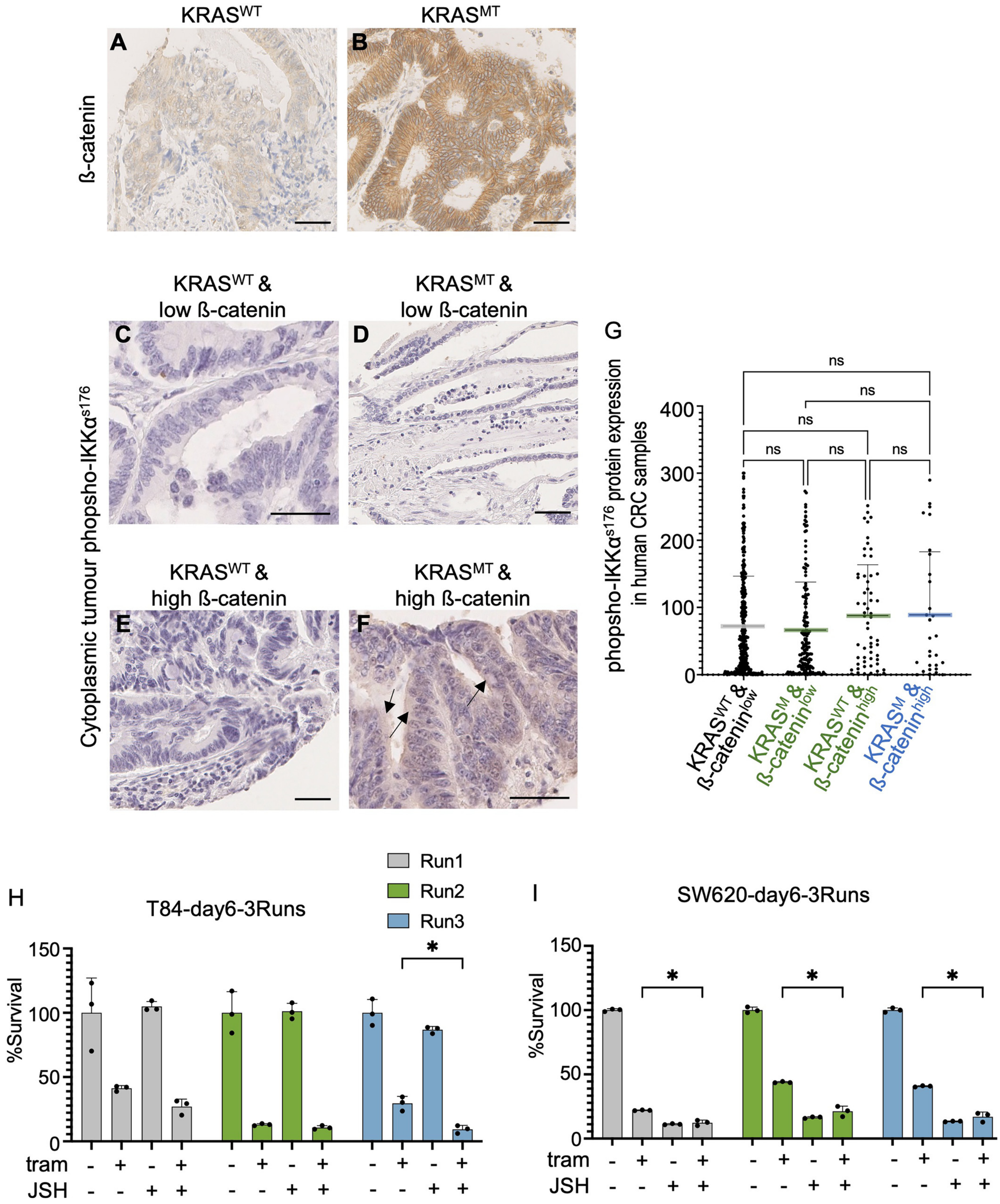
KRASWT
KRASMT
A
B
ß-catenin
Cytoplasmic tumour phopsho-IKKαs176
KRASWT &
low ß-catenin
KRASMT &
low ß-catenin
C
D
KRASWT &
high ß-catenin
KRASMT &
high ß-catenin
E
F
G
phopsho-IKKαs176 protein expression
in human CRC samples
ns
0
100
200
300
400
KRASWT &
ß-cateninlow
KRASM &
ß-cateninlow
KRASWT &
ß-cateninhigh
KRASM &
ß-cateninhigh
H
T84-day6-3Runs
Run1
Run2
Run3
I
SW620-day6-3Runs
%Survival
150
100
50
0
*
tram
JSH

Figure EV6. IHC staining of phospho-IKKα^{s176} protein in human CRC.

(**A–F**) IHC staining of β-catenin or phospho-IKKα^{s176} in each genotype CRC sample. (**A**) *KRAS^{WT}* (wild-type); (**B**) *KRAS^{MT}* (mutation in position G12/G13); (**C**) *KRAS^{WT}* plus low β-catenin; (**D**) *KRAS^{MT}* plus low β-catenin; (**E**) *KRAS^{WT}* plus high β-catenin; (**F**) *KRAS^{MT}* plus high β-catenin expression in CRC samples. Scale bar 50 μm. (**G**) The graph shows the mean of expression of phospho-IKKα^{s176} in each different mutated human CRC, determined by IHC intensity values. Patients were grouped into four categories based on KRAS status and β-catenin expression. Both *KRAS^{WT}* and β-catenin low ($n = 316$); *KRAS^{M}* and β-catenin low ($n = 164$); KRAS^{WT} and β-catenin high ($n = 59$); Both KRAS^{M} and β-catenin high ($n = 29$). Each dot displays an individual sample. (**H**, **I**) Cell viability assay for T84 or SW620 colon cancer cells, showing the results of three experimental replicates, treatment with 0.1% DMSO, 50 nM trametinib or 10 μM JSH-23. The statistical tests used to calculate the *P* value are as follows: (**G**) one-way ANOVA; (**H**, **I**) 2-way ANOVA; NS *P*(>0.12), **P*(0.033), ***P*(0.002) and ****P*(<0.001). All statistical data are summarised in Table EV1. The error bar is a standard deviation (SD), with each point representing biological replicates.

© The Author(s)
